# Stress keratin 17 enhances papillomavirus infection-induced disease by downregulating T cell recruitment

**DOI:** 10.1371/journal.ppat.1008206

**Published:** 2020-01-22

**Authors:** Wei Wang, Aayushi Uberoi, Megan Spurgeon, Ellery Gronski, Vladimir Majerciak, Alexei Lobanov, Mitchell Hayes, Amanda Loke, Zhi-Ming Zheng, Paul F. Lambert

**Affiliations:** 1 McArdle Laboratory for Cancer Research, University of Wisconsin, Madison, WI, United States of America; 2 Department of Dermatology, University of Pennsylvania, Philadelphia, PA, United States of America; 3 Tumor Virus RNA Biology Section, National Cancer Institute, Frederick, MD, United States of America; 4 CCR Collaborative Bioinformatics Resource (CCBR), National Cancer Institute, Bethesda, MD, United States of America; National Cancer Institute, UNITED STATES

## Abstract

High-risk human papillomaviruses (HPVs) cause 5% of human cancers. Despite the availability of HPV vaccines, there remains a strong urgency to find ways to treat persistent HPV infections, as current HPV vaccines are not therapeutic for individuals already infected. We used a mouse papillomavirus infection model to characterize virus-host interactions. We found that mouse papillomavirus (MmuPV1) suppresses host immune responses via overexpression of stress keratins. In mice deficient for stress keratin K17 (K17KO), we observed rapid regression of papillomas dependent on T cells. Cellular genes involved in immune response were differentially expressed in the papillomas arising on the K17KO mice correlating with increased numbers of infiltrating CD8^+^ T cells and upregulation of IFNγ-related genes, including CXCL9 and CXCL10, prior to complete regression. Blocking the receptor for CXCL9/CXCL10 prevented early regression. Our data provide a novel mechanism by which papillomavirus-infected cells evade host immunity and defines new therapeutic targets for treating persistent papillomavirus infections.

## Author summary

Persistent infection with high-risk human papillomavirus may lead to a variety of cancers in human including cervical cancer, anal cancer, head and neck cancer and skin cancer. There has been a lack of animal model to study how papillomavirus infection evades cellular immune surveillance to establish long-term infections to cause cancers. We utilized a mouse papillomavirus, MmuPV1, as an infection model in immunocompetent FVB/N mice to study how the virus escapes immune recognition to establish persistent infections. We found a novel regulator, stress keratin 17 (K17), was induced in expression following MmuPV1 infection, and that K17 was critical for preventing T cell infiltration in MmuPV1-induced papillomas by inhibiting the CXCL9/CXCL10/CXCR3 axis. Our study provides a new mechanism by which papillomaviruses evade T cell response following infection.

## Introduction

High-risk human papillomaviruses (HPV) cause 5% of all human cancers, including cervical cancer, head and neck squamous cell carcinoma (HNSCC), anal cancer and subtypes of skin cancer. Due to the species specificity of papillomaviruses, we and others have previously used HPV16 E6/E7 transgenic mice that express the high-risk HPV oncogenes E6 and E7 in epithelial tissues under the K14 promoter to study papillomavirus-related pathogenesis [1–4]. When combined with various co-factors for tumorigenesis, these mice have been informative tools to study the oncogenic roles and signaling pathways whereby HPV16 E6 and E7 contribute to skin cancer [2], head and neck cancer [5, 6], anal cancer [7, 8] and cervical cancer [4, 9, 10]. While these genetically engineered mice have contributed greatly to our understanding of neoplastic disease caused by papillomaviruses, they are not appropriate laboratory models to study host immune responses to papillomavirus-induced disease because thymic epithelial cells, wherein T cell selection occurs, also express E6 and E7, resulting in T cell tolerance to E6 and E7 [11–13]. Furthermore, the transgenic model is not suitable for modeling disease arising from natural papillomavirus infections in humans. These limitations have been overcome by the discovery of the mouse papillomavirus, MmuPV1 or MusPV1, in 2011 [14], the first papillomavirus discovered to infect laboratory mice (*Mus musculus*). We and others found that MmuPV1 can be used to model infection by high-risk HPVs: 1) MmuPV1 and HPVs encode a similar set of genes and express similar patterns of viral transcripts [15, 16]; 2) like HPV, MmuPV1 E6 and E7 genes both play critical roles in pathogenesis [17, 18] (manuscript in preparation); 3) MmuPV1 preferentially causes disease in immunocompromised hosts [19–22] as observed for HPVs in humans; and 4) persistent viral infections lead to cancer in both cutaneous and mucosal sites [20, 23–25]. Prior studies of MmuPV1 infection in immunocompetent FVB/N mice showed that persistence of cutaneous papillomas correlated with an immunosuppressive state [20]. However, how MmuPV1 infection modulates immune surveillance and the host factors contributing to this process is unknown. In this report, we identified a new host factor, stress keratin 17, which contributes to MmuPV1 infection-induced cutaneous papillomatosis by modulating the host cellular immune response to MmuPV1 infection.

## Results

### Stress keratin 17 was upregulated in MmuPV1-induced cutaneous papillomas and was required for persistent papilloma growth

**Fig 1:**
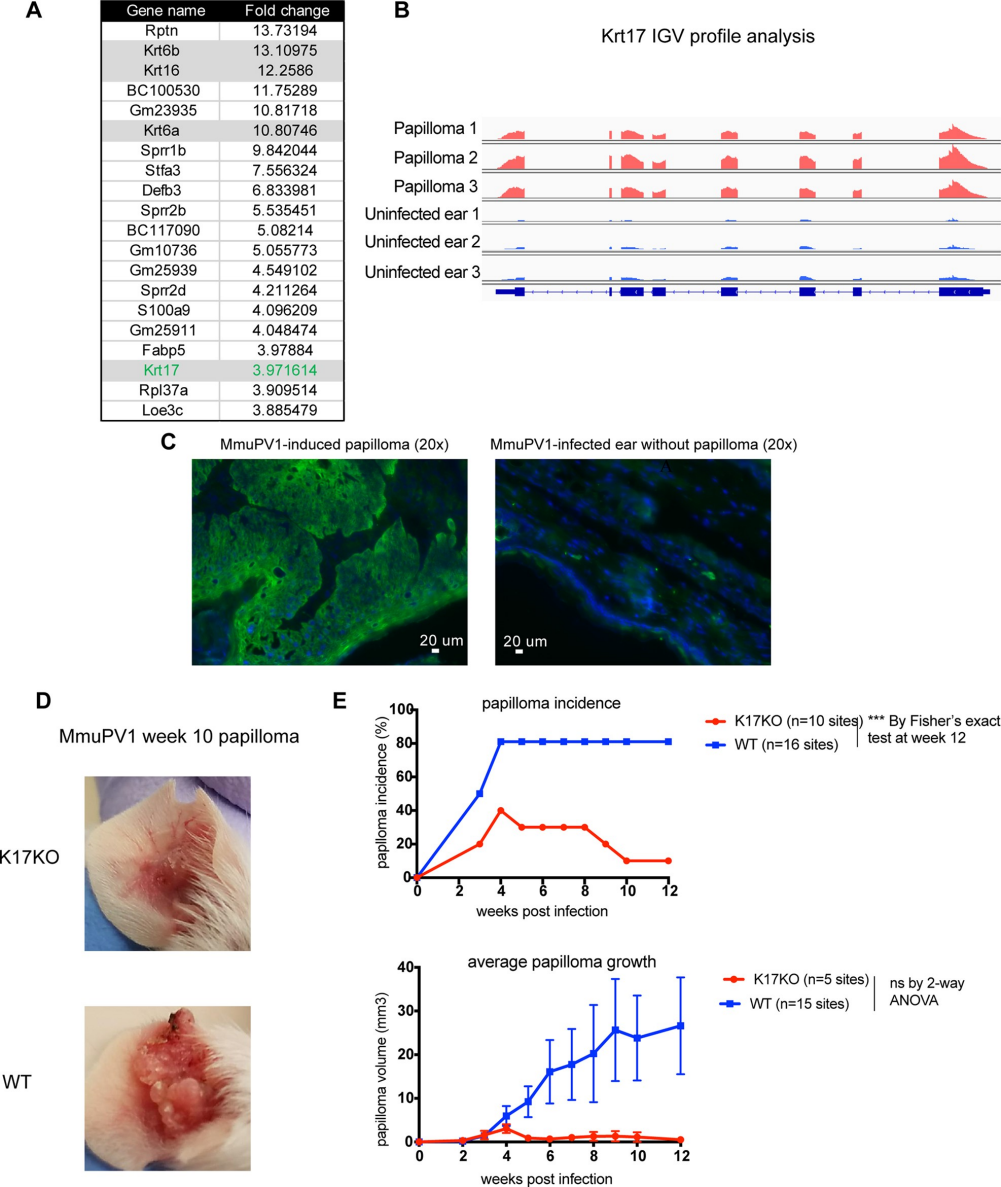
Stress keratin17 was up-regulated in MmuPV1-induced papilloma and was required for papilloma persistence in FVB/N mice. A) RNA sequencing data showing top 20 up-regulated genes by fold change in MmuPV1-induced papillomas in BALBc *FoxN1*^*nu/nu*^ mice. Stress keratins (Krt6a, Krt6b, Krt16, Krt17) are highlighted in grey. Fold change was calculated using three papilloma samples compared to three ear samples shown in Fig 1B. B) RNA-seq reads coverage mapped to Krt17 gene in each sample visualized by IGV with the equal scale of maximal RNA reads (0–8941) for all samples. C) Immunofluorescent staining for keratin 17 (green) in MmuPV1-induced wart (left) or normal ear (right) tissues, counterstained with DAPI (blue). D) Representative images of papillomas present on K17KO (top) and WT (bottom) mouse ears 10 weeks following infection with 10^9^ VGE/site MmuPV1 (Prep#1). E) Papilloma incidence (top graph) and size (bottom graph) arising in K17KO (red lines) versus WT (blue lines) mice. Data are represented as mean ± SEM. Fisher’s exact test was used to compared papilloma incidence for each time point, p value not adjusted. 2-way ANOVA Sidak multiple comparison was used to compared papilloma size. ***p<0.005; ns = not significant.

To identify host factors that are dysregulated during MmuPV1 infection, we analyzed RNA seq data from MmuPV1-induced ear papillomas versus uninfected normal ear tissues from BALB/c *FoxN1*^*nu/nu*^ (nude) mice. Among the top upregulated genes in MmuPV1-induced papillomas were a gene family called stress keratins (Fig 1A, S1 Table). Stress keratins, including keratin 6a (Krt6a or K6a), keratin 6b (Krt6b or K6b), keratin 16 (Krt16 or K16) and keratin 17 (Krt17 or K17), are undetectable in the inter-follicular epidermis of normal skin but are upregulated upon stress stimuli such as wound healing [26]. Integrative Genomics Viewer (IGV) profiling of stress keratin RNA reads showed abundant reads mapping to these genes in papilloma samples arising in *FoxN1*^*nu/nu*^ mice compared to uninfected normal ear tissues (Fig 1B, S1 Fig). Interestingly, two labs independently reported that stress keratins are upregulated in both the cutaneous and mucosal epithelial lesions in HPV16 transgenic mice [27, 28], suggesting these genes may be involved in HPV-induced disease. We verified overexpression of one of these stress keratins, K17, at the protein level using immunofluorescence in MmuPV1-induced papillomas arising in immunocompetent FVB/N mice (Fig 1C).

To test whether expression of K17 was required for MmuPV1-induced papillomatosis, we obtained mice in which the K17 gene is knocked out on the FVB/N genetic background (K17KO) [29]. We infected scarified ears of K17KO and WT FVB/N mice with MmuPV1, and monitored papilloma incidence and growth. MmuPV1 infection induced papillomas in both groups of mice, but did so more efficiently at the infected sites of WT FVB/N mice than on the K17KO mice (Fig 1D and 1E). Moreover, the papillomas that developed on WT mice continued to grow over the nine-week duration of the experiment; whereas, MmuPV1-infected sites on K17KO mice developed fewer papillomas and those papillomas quickly shrank in size after 4 weeks (Fig 1D and 1E). These data indicate that K17 is required for efficient and continued papilloma growth in immunocompetent FVB/N mice.

### Papilloma regression in K17KO mice was T cell-dependent

**Fig 2:**
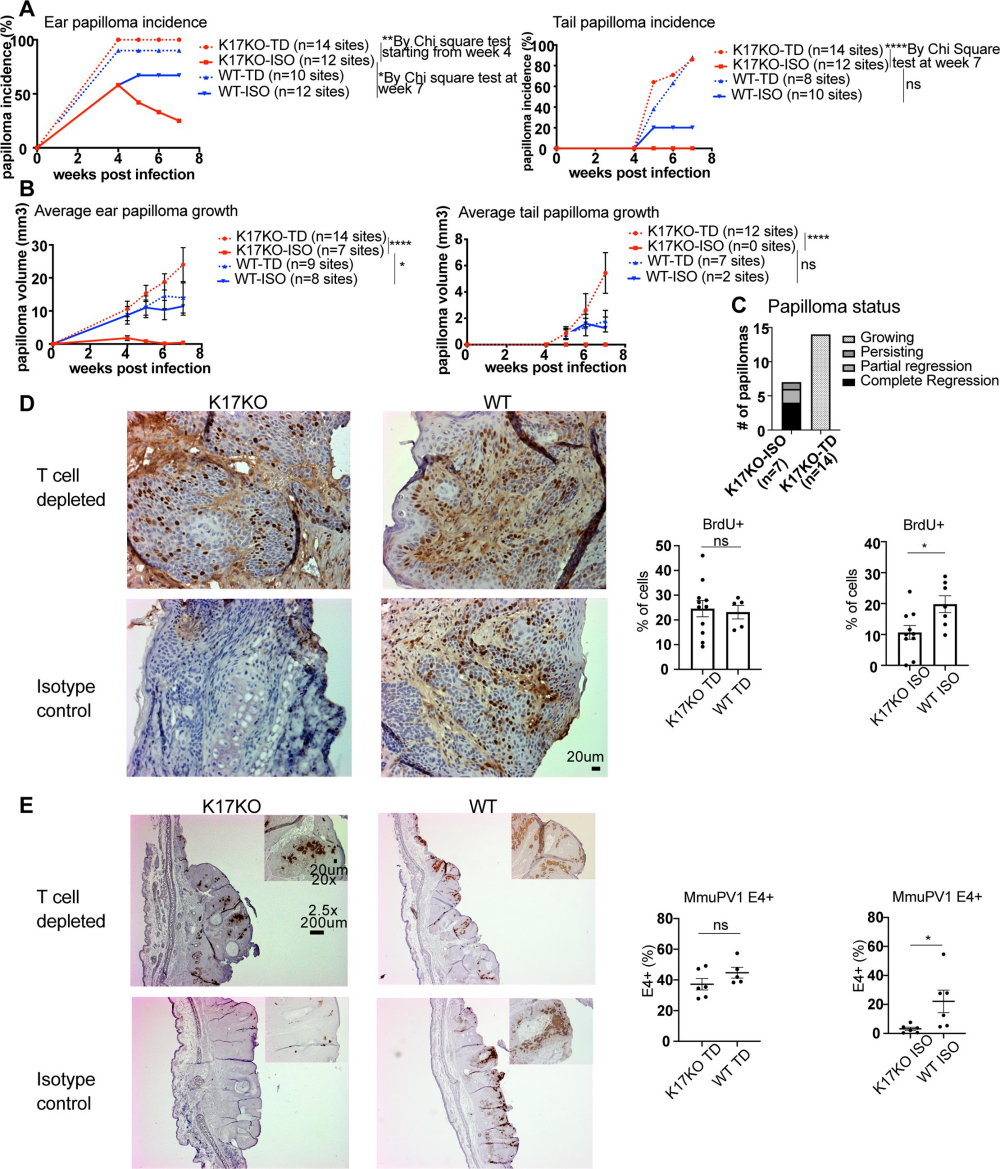
T cells are required for the early papilloma regression in K17KO mice. Mouse ears and 2 tail sites per mouse were infected with 2x10^8^ VGE MmuPV1 (Prep#2) per site, with both CD4 and CD8 T cell depletion (TD) or an isotype antibody (ISO) injection as a control starting 4 days prior to infection throughout the study. A) Papilloma incidence and average growth on ears and tails of WT (blue) and K17KO (red) in ISO control (solid) versus T cell-depleted (dashed) mice. *p<0.05, **p<0.01, ****p<0.001. Chi square test was used to compare papilloma incidence at each time point (p value not adjusted). B) Average papilloma growth on ears and tails of WT (blue) and K17KO (red) in ISO control (solid) versus T cell-depleted (dashed) mice. *p<0.05, ****p<0.001, 2-way ANOVA Dunnett’s multiple comparison test was used to compare papilloma size. C) Papilloma growing status between week 4 and week 7 in K17KO ISO control mice and T cell-depleted mice. D) BrdU (IHC) and hematoxylin staining of three representative images (20x) from each group. One field (20x) was evaluated from each ear papilloma. All images were quantified using an automated counting macro for ImageJ. Data are represented as mean ± SEM. Student t test was used. *p<0.05; ns = not significant. E) MmuPV1 E1^E4 transcripts were detected by in situ RNAscope, hematoxylin was used for counterstaining. One representative papilloma or regressed site are shown for each group (2.5x). 20x image is shown in upper right corner. One field (20x) was evaluated from each sample. All images were quantified using an automated counting macro for ImageJ. Data are represented as mean ± SEM. Student t test was used. *p<0.05; ns = not significant.

We hypothesized that the early papilloma regression in K17KO mice could be mediated by immune responses and/or deficiency in cell proliferation and MmuPV1 viral gene expression. Since stress keratin 17 has been implicated in regulating Th1/Th2 cytokine profiles in lesions arising in HPV16 transgenic mice [27, 30], we tested whether a cellular immune response was necessary for papilloma regression in K17KO mice. We depleted both CD4^+^ and CD8^+^ T cells starting 4 days before MmuPV1 infection on ears and tails and then continuing depletion until the endpoint of 7 weeks post-infection while following papillomatosis in both K17KO and WT mice. We confirmed the absence of T cells in circulating blood and papillomas in the T cell-depleted animals by flow cytometry (S2 Fig). In K17KO mice treated with isotype control antibodies, 7 out of 12 (58%) infected ear sites established cutaneous papillomas, while in T cell-depleted K17KO mice 14 out of 14 (100%) infected ear sites developed papillomas (Fig 2A and 2B). Four out of 7 established papillomas in K17KO mice treated with isotype control completely regressed by week 7, 2 out of the 7 established papillomas underwent partial regression, and 1 papilloma persisted (Fig 2C). In contrast, 14 out of 14 established papillomas in T cell-depleted K17KO mice continued to grow (Fig 2C). Two tail sites were also infected on these same animals, and the papilloma incidence and growth on tail sites were consistent with what we observed on ear sites (Fig 2A and 2B). In WT and K17KO animals with T cells depleted, K17KO mice developed as many papillomas as WT mice on infected ears and tails (Fig 2A). The sizes of the papillomas arising in T cell-depleted K17KO mice were comparable to those arising in WT mice (Fig 2B). These data suggest that MmuPV1 infection of cutaneous tissue in K17KO mice leads to efficient papillomatosis and papilloma growth in the absence of T cells.

To confirm that MmuPV1-infected K17KO keratinocytes are not inherently inefficient at proliferating or inefficient at supporting MmuPV1 viral gene expression (i.e. there is not a cell autonomous effect of K17KO status within epithelial cells), papillomas arising from T cell-depleted animals were evaluated for BrdU incorporation and MmuPV1 E1^E4 transcript expression at the endpoint of T cell depletion study. In the T cell-depleted animals, K17KO papillomas had similar frequencies of BrdU^+^ keratinocytes in the papillomas compared to papillomas arising on WT mice (Fig 2D) as well as abundant expression of MmuPV1 E1^E4 transcripts (Fig 2E). In contrast, in control K17KO papilloma (K17KO isotype control), there was a lower frequency of BrdU^+^ keratinocytes (Fig 2D) and barely any detectable MmuVP1 E1^E4 transcript expression compared to control WT papilloma (WT isotype control) (Fig 2E). These data suggest that K17KO keratinocytes have similar susceptibility to MmuPV1 infection as WT keratinocytes and that MmuPV1 viral gene expression was not interrupted in K17KO keratinocytes, since they had similar incidence of papillomas and similar expression of viral E1^E4 transcripts when T cells were absent. Therefore, the rapid regression of papillomas, reduced proliferation of cells and downregulation of viral transcription in K17KO mice is dependent upon T cell surveillance.

### Immune response genes were upregulated in MmuPV1-induced papillomas that developed on K17KO mice compared to WT mice

**Fig 3:**
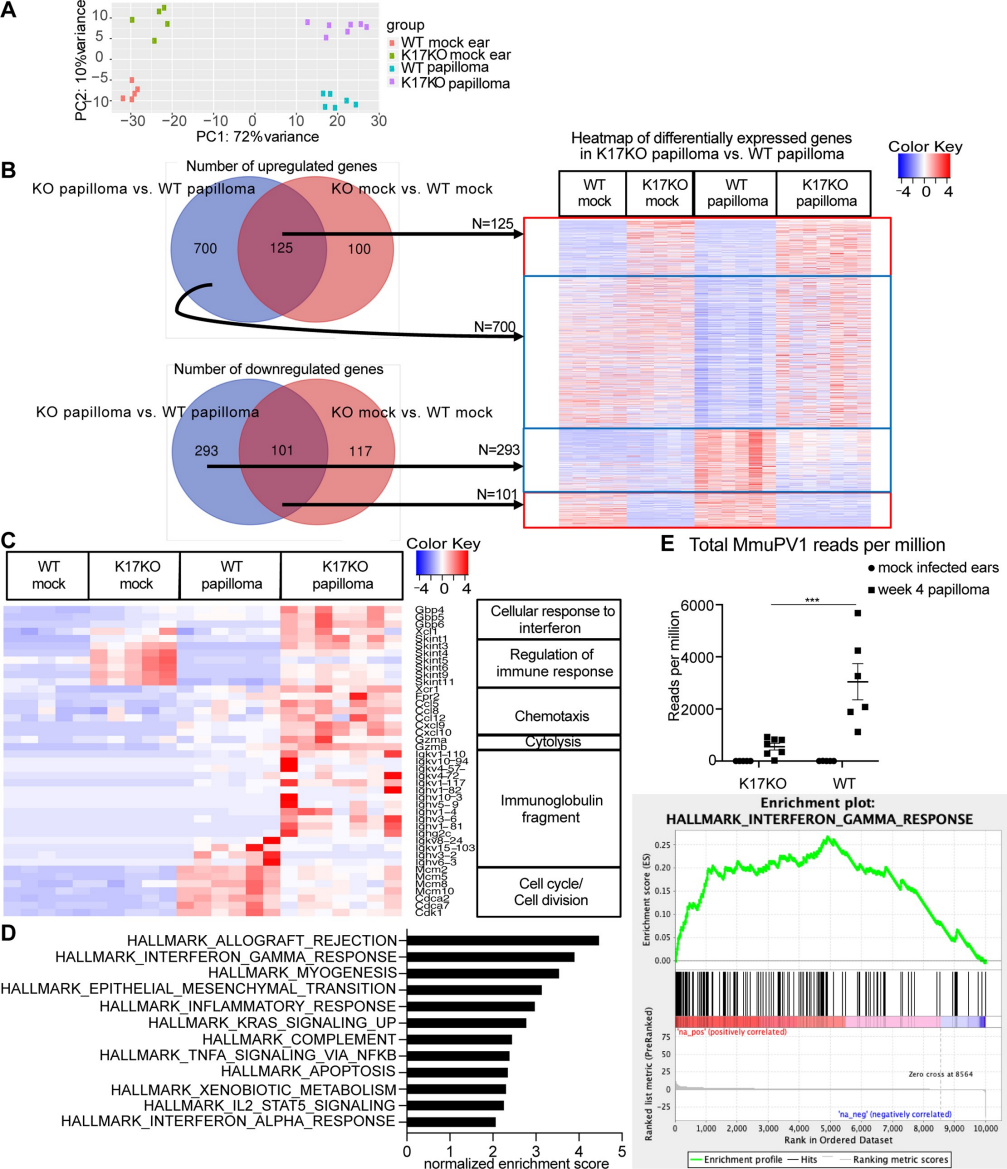
Systematic unbiased gene expression analysis revealed that the top upregulated pathways in K17KO papilloma were related to immune response. Mouse ears were infected with 10^9^ VGE MmuPV1(Prep#1) per site. A) Principal Component Analysis (PCA) clustering of four groups of samples based on RNA-seq data from FVB/N mice. B) Heatmap of all significantly upregulated and downregulated (adjusted p-value <0.05 and fold change >1.5) genes between K17KO papilloma and WT papilloma samples. C) Heatmap of representative genes in gene ontology analysis between WT papilloma and K17KO papilloma. D) Gene set enrichment analysis (GSEA) was done with genes that were determined to have a human gene homolog by Ensembl. Normalized enrichment score for top 12 enriched upregulated gene sets and enrichment plot for interferon gamma response gene set are shown. E) Total reads mapped to MmuPV1 genome normalized to total reads mapped to mouse host genome. Data are represented as mean ± SEM. 2-way ANOVA Sidak multiple comparison was used to compared viral transcripts. ***p<0.005.

To investigate which immune-regulating genes were involved in early regression of papillomas in K17KO mice, we harvested K17KO and WT papillomas at 4 weeks post-infection, and analyzed differentially expressed genes and pathways between K17KO and WT papillomas by RNA-seq analysis in four groups of mice: 1) mock-infected WT FVB/N mice, 2) mock-infected K17KO FVB/N mice, 3) papillomas arising on MmuPV1-infected WT FVB/N mice at 4 weeks post-infection, and 4) papillomas arising on MmuPV1-infected K17KO FVB/N mice at 4 weeks post-infection. Principal component analysis (PCA) showed distinct clustering of these four groups (Fig 3A), indicating distinct gene expression patterns. There were 825 significantly upregulated host genes and 394 significantly downregulated host genes in K17KO papillomas compared to WT papillomas (defined by adjusted p-value< 0.05 and fold change > 1.5 or < 0.67) (Fig 3B, S2 Table). Of the 825 upregulated genes, only 125 genes were also upregulated in the absence of MmuPV1 infection due to K17 gene deletion (K17KO mock vs. WT mock). Of the 394 downregulated genes, 101 genes were also downregulated in the absence of MmuPV1 (K17KO mock vs. WT mock) (Fig 3B). Gene ontology analysis for upregulated and downregulated genes in K17KO papilloma vs. WT papilloma was done with GeneCodis using *Mus musculus* genes as the reference list (GeneCodis is a web-based tool using an integrative and modular enrichment analysis [MEA] approach that takes into account interactions between proteins and dependencies between genes) [31–34]. Interestingly, most upregulated genes in K17KO papillomas were categorized as immune response-related genes (Table 1, for full GO list, see S3 Table). Among these immune-related genes, a family called “selection and upkeep of intraepithelial T-cells proteins”, or *Skint*, genes [35, 36] were also upregulated in K17KO mock ears (vs. WT mock ears) (Fig 3C). This subset of differentially expressed genes may reflect intrinsic differences between K17KO and WT tissues, or genes that are induced by wounding (mock infection) alone, in the absence of MmuPV1 infection.

**Table 1:** Gene Ontology analysis of differentially expressed genes in K17KO papilloma vs. WT papilloma.

**Top five GO gene sets that were upregulated in K17KO papilloma (vs. WT papilloma) and upregulated in K17KO mock ear (vs. WT mock ear)**						
**Annotations**	**NGR**^**a**^	**TNGR**^**b**^	**NG**^**c**^	**TNG**^**d**^	**Hyp**^**e**^	Hyp*^f^
GO:0035458: cellular response to interferon-b (BP)	38	37681	8	139	1.25E-12	7.54E-10
GO:0050776: regulation of immune response (BP)	56	37681	7	139	1.59E-09	3.21E-07
GO:0042832: defense response to protozoan (BP)	30	37681	6	139	1.25E-09	3.77E-07
GO:0050852: T cell receptor signaling pathway (BP)	90	37681	7	139	4.62E-08	6.99E-06
GO:0006952: defense response (BP)	103	37681	6	139	2.41E-06	0.000292
**Top five GO gene sets that were upregulated in K17KO papilloma (vs. WT papilloma) but not differentially expressed in K17KO mock ear (vs. WT mock ear)**						
**Annotations**	**NGR**^**a**^	**TNGR**^**b**^	**NG**^**c**^	**TNG**^**d**^	**Hyp**^**e**^	Hyp*^f^
GO:0006954: inflammatory response (BP)	313	37681	26	488	9.14E-14	1.65E-10
GO:0002376: immune system process (BP)	434	37681	27	488	2.70E-11	2.45E-08
GO:0030198: extracellular matrix organization (BP)	151	37681	15	488	1.41E-09	5.10E-07
GO:0006935: chemotaxis (BP)	126	37681	14	488	1.14E-09	5.16E-07
GO:0035458: cellular response to interferon-b (BP)	38	37681	9	488	1.11E-09	6.72E-07
**Top five GO gene sets that were downregulated in K17KO papilloma (vs. WT papilloma) but not differentially expressed in K17KO mock ear (vs. WT mock ear)**						
**Annotations**	**NGR**^a^	**TNGR**^b^	**NG**^c^	**TNG**^d^	**Hyp**^e^	Hyp*^f^
GO:0007049: cell cycle (BP)	611	37681	43	212	3.56E-34	3.14E-31
GO:0051301: cell division (BP)	370	37681	33	212	1.30E-29	5.76E-27
GO:0007059: chromosome segregation (BP)	83	37681	15	212	9.42E-19	2.77E-16
GO:0000278: mitotic cell cycle (BP)	130	37681	13	212	5.73E-13	1.27E-10
GO:0000070: mitotic sister chromatid segregation (BP)	28	37681	8	212	2.48E-12	4.38E-10

^a^NGR = Number of annotated genes in the reference list

^b^TNGR = Total number of genes in the reference list

^c^NG = Number of annotated genes in the input list

^d^TNG = Total number of genes in the input list

^e^Hyp = Hypergeometric pValue

^f^Hyp* = Corrected hypergeometric pValue.

Many of the upregulated genes in K17KO versus WT MmuPV1-induced papillomas were not upregulated in K17KO mock-infected ears, including many chemokines for immune cell chemotaxis (such as *CCL5*, *CCL8*, *CXCL9* and *CXCL10*), T and NK cell mediated-cytotoxicity (*Gzma*, *Gzmb*) and immunoglobulin heavy chain and kappa chain variable region sequences (Fig 3C). These differentially expressed genes are presumably regulated by MmuPV1 infection and indicate that, following MmuPV1 infection, the microenvironment of K17KO papillomas is enriched for cell-mediated immune responses, and one that may perhaps indicate a better humoral response.

In addition, gene ontology analysis also revealed that the genes that were significantly downregulated in K17KO papillomas (vs. WT papillomas) were related to cell cycle and cell division (Table 1, Fig 3C). This was consistent with our observation of smaller papillomas with fewer BrdU+ cells in K17KO mice compared to WT mice (Fig 2B and 2D) when T cells are present.

We also performed gene set enrichment analysis (GSEA) with unfiltered gene expression datasets. All mouse gene names were converted into human gene names, and only those mouse genes with a human homolog were included for GSEA analysis. Hallmarks gene sets (h.all.v6.2.symbols.gmt) were used as the gene set database. GSEA analysis showed that 15 out of 46 upregulated gene sets were significantly enriched at nominal p value < 1%; no downregulated gene sets were significantly enriched at nominal p value <1% (S4 Table). The top enriched upregulated gene sets were associated with allograft rejection and interferon gamma response (S5 Table, Fig 3D), consistent with the gene ontology analysis (Table 1).

RNA-seq reads were also mapped to the MmuPV1 genome to confirm the presence of viral transcripts in MmuPV1-induced papillomas. Consistent with our observation that papillomas in MmuPV1-infected K17KO mice regressed soon after 4 weeks post-infection (Figs 1E and 2A), and that control K17KO papillomas (K17KO isotype control) had low levels of E1^E4 transcripts detected by RNA scope (Fig 2E), we observed a lower number of RNA reads mapping to the MmuPV1 genome in the K17KO papillomas compared to WT papillomas (Fig 3E). These data indicate that K17 regulates global gene expression in MmuPV1-induced papillomas, and specifically genes associated with the host immune response and cell cycle/cell division.

### Lack of stress keratin 17 leads to increased infiltration of CD8^+^ T cells and IFNγ-related gene expression

**Fig 4:**
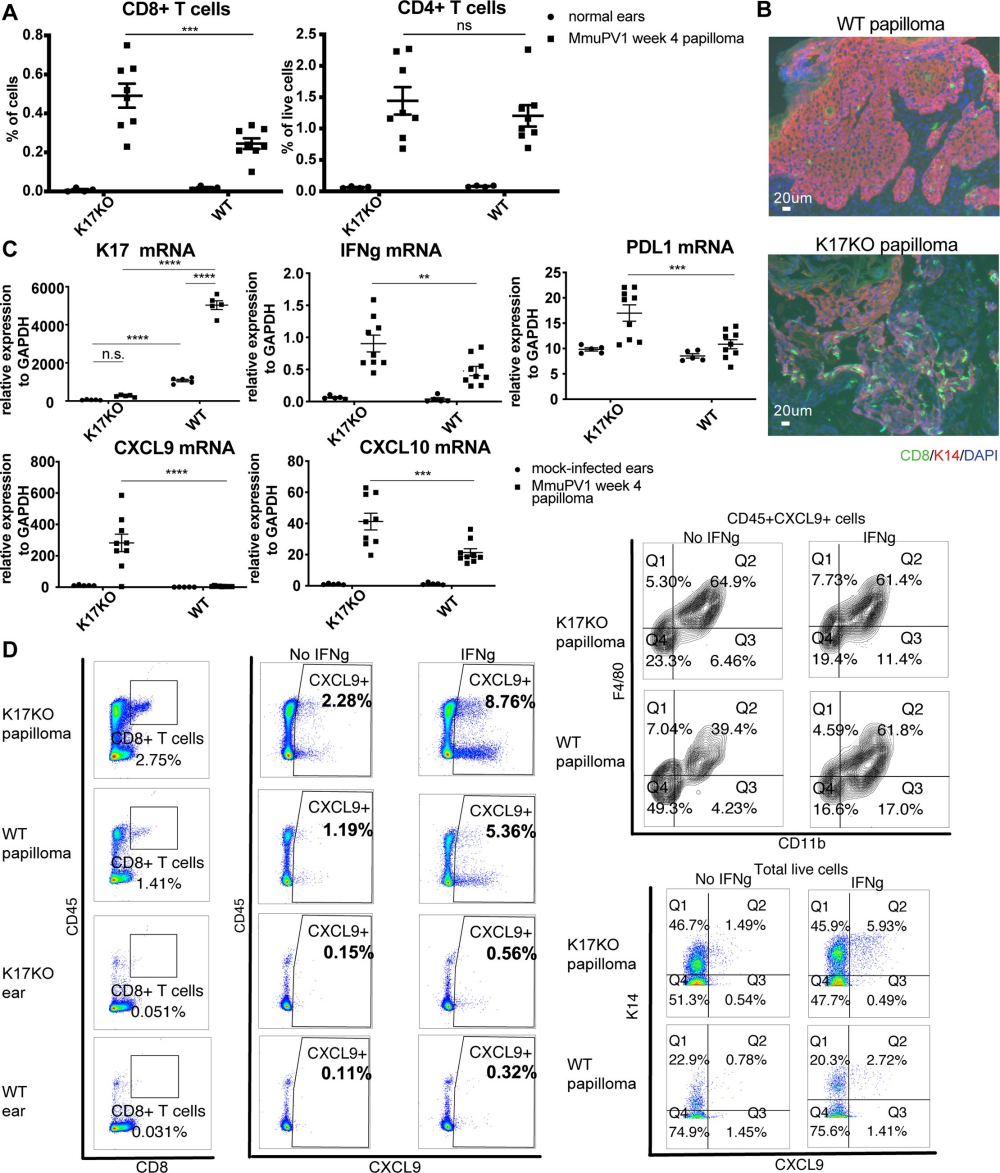
Lack of stress keratin 17 leads to increased infiltration of CD8^+^ T cells and increased expression of IFNg-related genes. Mouse ears were infected with 10^9^ VGE MmuPV1(Prep#1) per site. A) Percentage of CD8^+^ and CD4^+^ T cells based on flow cytometry analysis of normal ears (circles) versus ear MmuPv1 papillomas (squares) 4 weeks post-infection. For quantification of other populations, see S4A Fig. B) Immunofluorescent staining of frozen tissue sections of ear papillomas at 4 weeks post-infection. Green: CD8; Red: keratin 14 (K14); Blue: DAPI. Representative image for n = 4 samples (K17KO) and n = 6 samples (WT). C) RT-qPCR analysis of bulk RNA in mock-infected ears (circles) and ear MmuPV1 papillomas (squares) from K17KO and WT tissue 4 weeks post-infection. K17 expression was measured by TaqMan probe. IFNγ, PDL1, CXCL9 and CXCL10 were measured by SYBR Green method. GAPDH was used for normalization. Data are represented as mean ± SEM. 2-way ANOVA Sidak multiple comparison was used. **p<0.01; ***p<0.005; ****p<0.001; ns = not significant. D) Week 4 papilloma and uninfected normal ears were harvested from K17KO and WT FVB/N mice. Single cell suspension was cultured with or without recombinant mouse IFNγ (1000 U) in the presence of protein transport inhibitor for 16 hours. Cells were washed and stained with GhostDye to exclude dead cells, then stained with cell surface marker and intracellular CXCL9. A small aliquot of each sample was stained with K14 and CXCL9 only. (Left panel) Both CD45^+^ and CD45^-^ cells could produce CXCL9 from papilloma tissues, with CD45^-^ cells being the major population. (Top right panel) Within CD45^+^ CXCL9^+^ cells, majority of the cells were CD11b^+^F4/80^+^ macrophages. (Bottom right panel) Majority of the CXCL9-producing cells were stained K14^+^. Representative sample is shown for n = 3 K17KO papilloma and n = 3 WT papilloma. Only one K17KO ear and one WT ear were included as control.

Since many of the most significantly upregulated pathways in K17KO papillomas were associated with immune responses (Table 1, Fig 3C), we measured infiltrating immune populations by flow cytometry as well as IFN gamma (IFNγ)-related gene expression levels by RT-qPCR in K17KO and WT papillomas. When we characterized immune infiltrates by flow cytometry in WT papillomas at 7 weeks post-infection, we found some immune population frequencies could vary by the growth status of the papillomas (actively growing versus regressing) (S3 Fig). Therefore, we harvested papillomas from K17KO mice at the peak of papillomatosis and compared them to WT papillomas harvested at the same time. We found increased CD8^+^ T cells in K17KO papillomas, while differences in levels of other immune cells including CD4^+^ T cells were not significant (Fig 4A and S4A Fig). The increased infiltration of CD8^+^ T cells in K17KO papillomas versus WT papillomas was confirmed by histological staining (Fig 4B). We also confirmed upregulated expression of IFNγ-related genes by RT-qPCR, including *IFN*γ, *PD-L1*, *CXCL9* and *CXCL10* in K17KO papillomas (Fig 4C). We also evaluated the percentage of activated CD8^+^ T cells as well as Th1 and Th2 cells in papilloma-draining lymph nodes, but we did not detect differences in the frequency of activated T cells in draining lymph nodes (S4B Fig). These results suggest that the increased CD8^+^ T cell infiltration in K17KO papillomas was due to increased chemotaxis mediated by CXCL9 and CXCL10, rather than increased expansion of activated T cells in the draining lymph nodes. Flow cytometry analysis from papillomas at 4 weeks post-infection showed that the main CXCL9-producing cells were CD11b^+^F4/80^+^ macrophages and K14^+^ keratinocytes in both K17KO and WT papillomas (Fig 4D). These observations indicate that a lack of K17 leads to increased CD8^+^ T cell infiltration in MmuPV1-induced papillomas.

### Blocking CXCR3 abrogates the difference in papillomatosis in WT vs K17KO mice

**Fig 5:**
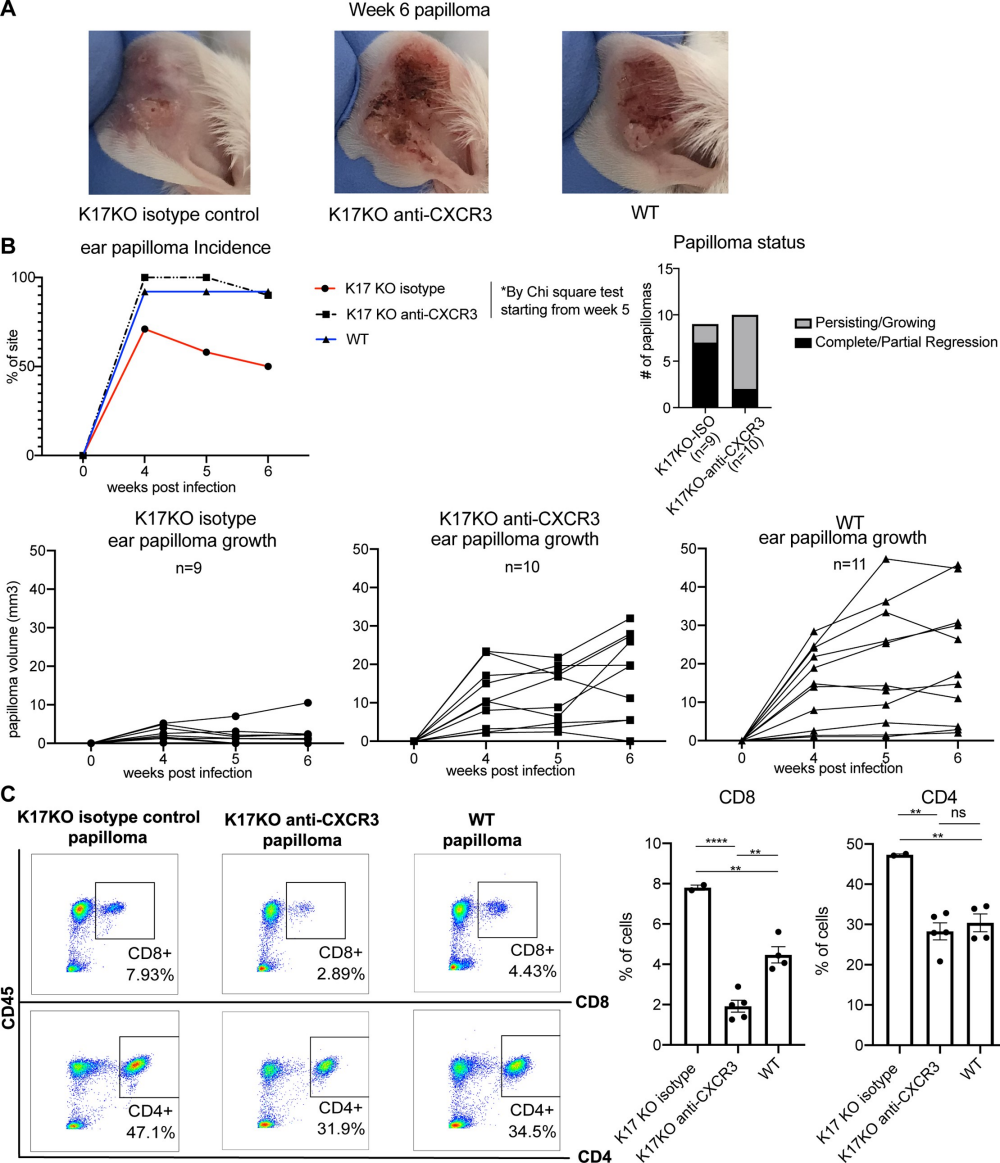
Blocking CXCR3 abrogates the difference between WT and K17KO papillomatosis. Mouse ears were infected with 2x10^8^ VGE MmuPV1 (Prep#2) and treated with an anti-CXCR3 or an isotype antibody as a control. A) Representative pictures of papillomas 6 weeks post-infection from isotype control treated K17KO, anti-CXCR3 treated K17KO and untreated WT FVB/N mice; B) Papilloma incidence (top left) and papilloma status (top right) on K17KO isotype control mice (n = 14 infected sites, red), K17KO anti-CXCR3 treated mice (n = 12 infected sites, dashed black) and untreated WT FVB/N mice (n = 12 infected sites, blue) infected with 2x10^8^ VGE/site on ears; (Bottom) Individual papilloma growth curves in K17KO mice treated with isotype control antibody or anti-CXCR3, and untreated WT mice. C) Flow cytometry analysis of infiltrating CD8^+^ and CD4^+^ T cells at 6 weeks post-infection in papillomas from K17KO and WT mice following anti-CXCR3 or isotype control treatment. Data are represented as mean ± SEM. One-way ANOVA Tukey's multiple comparisons test was used. *p<0.05; **p<0.01; ****p<0.001; ns = not significant.

Because CXCL9 and CXCL10 have well-established roles in recruiting CXCR3^+^ T cells into peripheral tissues under inflammatory conditions [37–41], and because we did not find a difference in Th1/Th2 in papilloma-draining lymph nodes between K17KO and WT mice (S4B Fig), we tested whether recruitment of T cells into the papilloma and early regression in K17KO mice was mediated by the CXCL9/CXCL10/CXCR3 signaling axis. We blocked CXCR3 in K17KO mice using anti-CXCR3 blocking antibody, starting 4 days before infection and continuing until 6 weeks post-infection, and followed papilloma growth. The absence of CXCR3^+^ cell detection in the blood following antibody injection was confirmed by flow cytometry (S5 Fig). The CXCR3 blocking antibody increased the incidence as well as the size of papillomas in the K17KO mice to levels comparable to that observed in the WT mice (Fig 5A and 5B). Ten out of 14 infected ear sites (71%, Fig 5B, red line) developed papilloma at 4 weeks post-infection in K17KO mice treated with isotype control while 12 out of 12 infected ear sites developed papilloma in K17KO treated with anti-CXCR3 antibody at 4 weeks post-infection (100%, Fig 5B, dashed black line). Individual papilloma growth curves showed that most papillomas arising in K17KO isotype control-treated mice experienced partial or complete regression between week 4 and week 6 post-infection (Fig 5B, lower left graph). In contrast, most papillomas in K17KO mice treated with anti-CXCR3 persisted or grew between week 4 and week 6 (Fig 5B, right bar graph and lower middle graph). These papilloma growth curves were similar to the growth curves from WT papillomas (Fig 5B, lower right graph). At 6 weeks post-infection, we quantified infiltrating CD4^+^ and CD8^+^ T cells in the remaining papillomas that did not completely regress in K17KO control mice, papillomas in K17KO mice blocked with anti-CXCR3 and papillomas in untreated WT mice. There were decreases in both infiltrating CD4 and CD8 T cells in the papillomas arising in K17KO blocked with anti-CXCR3 (Fig 5C), suggesting the increased infiltration of CD8^+^ T cells in K17KO mouse papillomas was mediated by the CXCL9/CXCL10/CXCR3 signaling axis.

## Discussion

HPV infection is controlled by the immune system, as evidenced by increases in HPV infections in immunosuppressed/immunoincompetent individuals [42]. Existing HPV prophylactic vaccines that elicit a humoral immune response cannot clear pre-established papillomavirus infections, which requires cellular immune responses that recognize and kill infected cells. There are ongoing efforts to develop therapeutic vaccines that can elicit effective cellular immune responses [43–45]; however, their effectiveness in eliciting a clinical response has been poor. Here, we identify K17 as a host factor induced in papillomaviruses-associated disease that mediates an immune-suppressive state within infected tissue in a natural infection model for HPVs. We further show that, in a K17-deficient background, host cellular immune responses were competent to efficiently clear MmuPV1-induced papillomas. We propose that stress keratins and the signaling axes they regulate represent new targets for improving the therapeutic effect of immunotherapies for papillomavirus-induced diseases.

Stress keratin 17 is overexpressed in several types of human squamous cell carcinomas (SCC), and its overexpression is correlated with poor prognosis in SCC patients [46–50]. We evaluated RNA-seq data from TCGA and found *K17* expression level was inversely correlated with that for *CD8A* and *CXCL9* in a cohort of 513 patients with head and neck cancer (S6 Fig). Although no information about HPV status is available for these patients, this data is consistent with the hypothesis that overexpression of K17 negatively affects the infiltration of T cells in human cancers.

Moreover, how K17 expression is induced in various types of cancer is largely unknown. In a study led by Zhussupbekova et al, they found upregulated expression of all stress keratins (*KRT6A*, *KRT6B*, *KRT16 and KRT17*) in hyperplastic skin of K14HPV16E7 transgenic mice, but the expression was not induced in K14E7/Rb^ΔL/ΔL^ mice whose skin lacked hyperproliferation phenotype due to E7’s inability to bind to mutant Rb [26]. These authors propose that K17 overexpression in K14HPV16E7 transgenic skin is a consequence of HPV oncogene-induced hyperproliferation. In a non-HPV mouse model of skin hyperplasia, K17 was also found upregulated and responsible for early onset of Gli2-induced skin tumorigenesis [49]. Therefore it remains unclear whether, in the MmuPV1 infection model, K17 expression is induced directly by MmuPV1 infection or is an indirect consequence of MmuPV1 infection-induced hyperplasia. Further studies are required to address the mechanism by which K17 expression is induced in MmuVP1-induced diseases.

Besides K17, stress keratins Krt6 (K6) and Krt16 (K16) were also found upregulated in MmuPV1-induced lesions (S1 Fig), as well as in human pre-malignant hyperproliferative epithelium and the skin of HPV16E7 transgenic mice [26]. Mutations in *Krt17* and *Krt16* are associated with Palmoplantar Keratoderma (PPK) lesions (i.e. abnormal thickening of palm skins) in humans [50], and mice that lack the *Krt16* gene develop spontaneous lesions resembling human PPK [51]. In addition, topical TPA-induced hyperproliferation was more prominent in K16 knockout mice [52], suggesting distinct functions of K16 and K17. Certain damage-associated molecular pattern (DAMP) genes (such as *S100A7A*, *S100A8*, *TSLP*) were found upregulated in *in vitro* TPA-treated K16 KO keratinocytes but not in K17 KO keratinocytes [52]. In our RNA seq data, we found no significant differences in expression of *Krt16 (p = 0*.*34)*, *S100A7A* (p = 0.25), *S100A8* (p = 0.13) or *TSLP* (p = 0.08) between K17KO papillomas vs. WT papillomas. Thus, although K16 has been implicated in inflammatory response, K16 and K17 may function through different mechanisms. Whether K16 expression is important for MmuPV1-induced disease has not been examined.

In addition to its role as a cytoskeletal intermediate filament protein, K17 has also been reported to interact with other proteins including cell cycle inhibitor p27^KIP1^ [49], heterogeneous nuclear ribonucleoprotein K (hnRNP K) [51] and autoimmune regulator (Aire) [52]. Knockout of K17 in HPV16 transgenic mice reduced HPV16-induced disease progression or rendered the animals disease free of cervical and cutaneous lesions [27, 52]. Consistent with our observation, in the cervical model, mRNA analysis at one-month post estrogen implantation (to induce epithelial hyperplasia) showed higher expression of IFNγ, CXCL9 and CXCL10 in K17KO tissues. However, the differences in the expression of those genes were no longer present at 3 months post estrogen implantation. Instead, they observed higher Th2 and Th17 cytokine expression together with higher number of intraepithelial CD4, F4/80^+^ and CD11b^+^ cells [27]. In this HPV transgenic model, the HPV16 oncogenes are expressed under the control of the K14 promoter, which is active before birth, and their T cells are therefore likely tolerant to these viral antigens. Consistent with this hypothesis, an effect of K17KO on intraepithelial CD8^+^ T cells was not observed. Our data in MmuPV1-induced K17KO papillomas clearly support the hypothesis that there is a strong cellular immune response early during infection to clear MmuPV1-infected papillomas in K17KO mice. In our natural infection model, the regression window in K17KO mice was rapid, consistent with previous observations [27] that upregulated IFNγ-related genes were observed only one month post-implantation of estrogen. In a small number of MmuPV1-infected K17KO animals with persisting ear papillomas that did not regress, we observed elevated circulating CD11b^+^Gr1 high cells at 3 months post-infection compared to papilloma-free K17KO mice (S7 Fig). CD11b^+^Gr1 high cells are often referred to as granulocytic myeloid derived suppresser cells (gMDSCs or PMN-MDSCs) in late stage cancer models [53, 54]. While this observation needs to be validated in additional mice, and the known suppressive function of the CD11b^+^Gr1 high cells from papilloma-persisting K17KO animals needs to be evaluated, it is consistent with the observations using the cervical cancer model [27] that chronic disease may induce unwanted inflammation that contributes to disease persistence.

Chung *et*. *al*. showed that K17 can interact with hnRNP K to regulate expression of CXCR3 ligands in a TPA-treated human skin carcinoma line, A431 cells [51]. In contrast to our results, they saw downregulated expression of CXCR3 ligands when K17 expression was knocked down by shRNA. The differences between our models may explain our distinct observations: 1) A431 is an HPV-negative carcinoma cell line; therefore, overexpression of K17 was not induced by papillomavirus infection, while the K17 overexpression is induced by MmuPV1 infection in our model; and 2) overexpression of CXCR3 ligands in our model is induced by an immune microenvironment induced by MmuPV1 infection, which may involve different signaling pathways than mediated by TPA. Our RNA-seq data provides candidate genes that may account for the difference in the immune responses between K17KO and WT mice. For example, Guanylate Binding Protein 5 (GBP5) has been shown to have anti-viral functions in HIV-infected cells [55]. This gene was among the *Gbp* members that were upregulated in K17KO mock-infected ears. Another gene family, *Skint*, were also upregulated in K17KO mock infected ears. Two of these genes, *Skint3* and *Skint9*, play important roles in the crosstalk between epithelial and dendritic T cells [35, 36, 56]. One gene of the Skint family, *Skint 1*, was found responsible for selection and maturation of Vγ5Vδ1+ T cells in thymus [36]. Vγ5Vδ1+ T cells are unconventional T cells that can be found in peripheral intraepithelial compartment and can directly interact with epithelial cells. Since K17 expression has been detected in both adult and embryo thymic epithelial cells [57], it would be interesting to test whether there is also elevated *Skint 1* expression and increased abundance of mature Vγ5Vδ1+ T cells in the thymus of K17KO mice, regardless of MmuPV1 infection. Whether these intrinsic changes in K17KO mock-infected tissues in the absence of MmuPV1 are important for the upregulated immune response when mice are infected with MmuPV1 warrants further study. In addition, how K17 regulates the expression of these genes is unknown.

CXCR3-mediated T cell migration has been reported to be important to clear infections in other contexts, including infections by epicutaneous vaccinia virus [58], protozoan parasite *Toxoplasma gondii* [59], and herpes simplex virus type 2 [37]. In addition, Kuo et al. found elevated CXCL9 and CXCL10 levels in cutaneous lesions arising in HPV16 E7 transgenic mice and determined that CXCR3 is critical for HPV16 E7-expressing skin graft rejection [60]. CXCR3 is a receptor expressed on activated effector CD8+ T cells, NK cells, activated Th-1 CD4+ T cells and epithelial cells. There are three chemokine ligands for CXCR3: CXCL9, CXCL10 and CXCL11. Although these three ligands can have redundant functions, they have also been shown to function non-redundantly and regulate distinct responses [39, 61–63]. CXCL9 expression is solely induced by IFNg, while CXCL10 and CXCL11 can also be induced by type 1 IFNs. In the K17KO papillomas, while we found significant induction of CXCL9 and CXCL10, there was a significant reduction in CXCL11 RNA level compared to WT papillomas (p = 0.0002). When we blocked CXCR3, the papillomas in K17KO mice increased significantly in size, despite a reduction in CXCL11 level. Our findings that CXCL11 is expressed differentially from CXCL9/CXCL10 in the K17KO setting suggest that these chemokines are not regulated in the same manner and may play differential roles in some contexts.

In this report, we have shown that K17 overexpression in MmuPV1-induced papillomas supports persistent papilloma growth by suppressing the CXCL9/CXCL10/CXCR3 signaling axis and downregulating T cell recruitment to papillomas, indicating a novel role of K17 in contributing to MmuPV1 infection by affecting CXCR3 axis. Our data also suggest that K17 and its related signaling pathways serve as promising targets for improving efficacy of immunotherapies for papillomavirus-mediated disease.

## Materials and methods

### Animals

Wildtype FVB/N mice were obtained from Taconic and bred for this study as wildtype controls. K17 knockout (K17KO) mice on the FVB/N genetic background were provided by Pierre A Coulombe (Johns Hopkins University) and have been described previously [27]. All mice were housed in animal facility in aseptic conditions in micro-isolator cages and experiments carried out under an approved animal protocol. Six to eight week-old mice were used for experiments with an same ratio of male and females in each group. For T cell depletion experiment, 100 ug of anti-CD4 (BioXCell, clone GK1.5) and 100 ug of anti-CD8 antibody (BioXCell, clone 2.43) or 100 ug of isotype control (BioXCell, Rat IgG2b, κ) was delivered by intraperitoneal injection twice weekly, starting 4 days before MmuPV1 infection throughout the study. For detection of CD4 and CD8 depletion, CD8a FITC (Tonbo ebioscience, clone 53–6.7), CD4 PE (Tonbo ebioscience, clone RM4-5) were used for flow cytometry. For CXCR3 blocking experiment, 400ug of anti-CXCR3 (BioXCell, clone CXCR3-173) or isotype control antibody (BioXCell, Armenian Hamster IgG) was delivered i.p. three times a week, starting 4 days before MmuPV1 infection, throughout the study. This anti-CXCR3 clone is a well-established blocking antibody for CXCR3 in mouse studies [40, 64]. For detection of CXCR3 blocking in mice, the same clone was used (Biolegend, clone CXCR3-173).

### Ethics statement

Experiments were approved and performed in accordance with guidelines approved by the Association for Assessment of Laboratory Animal Care, at the University of Wisconsin Medical School. This study was approved by the University of Wisconsin School of Medicine and Public Health Institutional Animal Care and Use Committee under protocol number M005871.

### MmuPV1 isolation and infection

Crude preparations of MmuPV1 isolated from papillomas growing on nude mice were quantified for VGE (viral genome equivalents) per protocol described previously [20]. The infectivity of each MmuPV1 crude preparation (Prep#1 and Prep#2) was quantified and compared by *in vitro* infectivity assay in mouse JB6-clone 41 cells (gift from Dr. Nancy H. Colburn, NCI), using MmuPV1 E1^E4 transcript level as a readout [18] (S8 Fig). Based on *in vitro* infectivity of two preparations (S8 Fig), 10^9 VGE/ul of Prep#1 and 2x10^8 VGE/ul of Prep#2 were used respectively for separate *in vivo* infection experiments to achieve similar papilloma incidence in WT FVB mice. The same prep was used for all mice in the same experiment. Mouse ears were scarified and wounded using 27-gauge needle before infection. 2 ul of virus stock with indicated VGE in each figure legend were applied to the scarified ears. In mock infected mice, 2 ul of phosphate-buffered saline (PBS) was applied to scarified ears instead of MmuPV1 stock. Mock-infected animals were housed in separate cages and cage changing was performed on separate days from infected animals. Papilloma width, length and height were measured by caliper and used for calculation of papilloma size.

### Flow cytometry

Papillomas were trimmed of surrounding tissues and harvested on ice in PBS. Control ears or papillomas were cut into 1 mm pieces and digested in 5 mL HBSS supplemented with 5% fetal bovine serum (FBS), 2 mM CaCl_2_, 2 mM MgCl_2_, 1 mg/ml collagenase D (Roche) and 200U/ml DNase I (Roche), at 37˚C for 40 min. Tissues were then homogenized with the back of 1 ml syringe, passed through 0.7 μM filter and washed twice with cold PBS. Papilloma-draining lymph nodes were harvested and passed through 0.7 μM filter. Blood samples were collected from submandibular bleeding directly into red cell lysis buffer (Tonbo Biosciences) and incubated at room temperature for 10–15 min. Blood cells were then spun down and washed with PBS. Single cell suspensions were then stained with 1 ul Ghost Dye Violet 510 (Tonbo Biosciences) in 1 ml pf PBS at 4˚C for 30 min. Samples were then washed with PBS supplemented with 2% FBS and 0.1% NaN_3_, blocked with anti-mouse Fc receptor antibody and stained with cell surface markers. Cells were then washed and fixed with fixation buffer (eBioscience) overnight in 4 ˚C. Cells were washed in PBS supplemented with 2% FBS and 0.1% NaN_3_ and analyzed with ThermoFisher Attune. Flow cytometry beads (eBioscience) stained with each antibody were used as single-color controls. In studies where intracellular cytokines were evaluated, single cell suspensions were obtained from tissue and directly suspended in RPMI with 5% FBS and penicillin/streptomycin, in the presence of cell stimulation cocktail (eBioscience) and Golgi stop (eBioscience) for 16 hours and then stained with Ghost Dye and cell surface marker as described above. After fixation, cells were washed in permeabilization buffer (eBioscience) and stained in permeabilized buffer with antibodies detecting intracellular cytokines or proteins. A combination of selected antibodies (anti-mouse) was used depending on the purpose of each study: CD45 APC-Cy7 (Biolegend, clone 30-F11), CD8a FITC (Tonbo ebioscience, clone 53–6.7), CD4 PE (Tonbo ebioscience, clone RM4-5), Gr1 PE-Cy5 (Biolegend, clone RB6-8C5), F4/80 BV421 (Biolegend clone BM8), CD11b BV605 (Biolegend, clone M1/70), CD11c PE-Cy7 (Biolegend, clone N418), NKp46 BV711 (Biolegend, clone 29A1.4), IFNg PE-Cy7 (Biolegend, clone XMG1.2), IL4 BV421 (Biolegend, clone 11B11), IL17 BV711 (Biolegend, clone TC11-18H10.1), CXCL9 AlexaFluor647 (Biolegend, clone MIG-2F5.5), K14 purified (eBioscience, polyclonal Cat #PA5-16722), Donkey anti-rabbit AlexaFluor 488 (Invitrogen, cat #A21206).

### Immunofluorescent staining and immunohistochemistry

Fresh papillomas or ears were cut in half and embedded in optimal cutting temperature compound (OCT) and frozen on dry ice before storing in -80 ˚C. Frozen tissues were then sectioned (5 microns thick) using a cryostat. Tissue slides were fixed in cold methanol in -20 ˚C for 10 min, washed with PBS +0.01% Triton X-100, then pure PBS, blocked with 5% goat serum at room temperature for 1 hour, and stained with purified primary antibody at 4˚C overnight. Tissues were then washed with PBS three times, stained with secondary antibodies at room temperature for one hour, counterstained with Hoechst Dye and mounted in Prolong mounting media (Thermo Fisher Scientific). The following antibodies were used for detecting mouse antigens by immunofluorescent staining: CD4 (eBioscience, clone RM4-5), CD8 (eBioscience, clone 53–6.7), K14 (eBioscience, polyclonal Cat#PA5-16722), K17 (provided by Pierre A Coulombe [57]), Goat anti-rabbit AlexaFluor647 (Molecular Probes), Goat anti-rat AlexaFluor 488 (Molecular Probes). 4% paraformaldehyde (PFA) fixed tissues were used for 5-bromo-2'-deoxyuridine (BrdU) detection by immunohistochemistry. Each mouse was injected with 250 ul of 12.5 mg/ml BrdU in PBS i.p. one hour before euthanizing. Tissues were fixed in 4% PFA at 4 ˚C overnight and switched to 70% ethanol. They were then embedded in paraffin and sectioned into 5 microns pieces. Tissue sections were deparaffinized and blocked with 3% hydrogen peroxide. Antigens were retrieved in boiling 10 mM citrate buffer for 20 min. Tissues were then washed and stained overnight at 4 ˚C with anti-BrdU antibody (Millipore EMD, clone 131–14871). Anti-mouse HRP antibody and DAB (Vector M.O.M. Kit) were used to detect anti-BrdU antibody. Tissues were then counterstained with hematoxylin and mounted in Cytoseal media (Thermo Fisher Scietific). Quantification of total cells in epithelial or papilloma tissues and BrdU positive cells were done by an automatic counting program “BrdU Count v2.1.ijm” developed by Dr. David Ornelles (Wake Forest University). 4% PFA fixed tissues were used for MmuPV1 E4 transcript detection by RNAscope (Advanced Cell Diagnostics) followed by DNase I (Thermo Fisher Scientific) treatment as described previously [16]. Tissues were then counterstained with hematoxylin and mounted in Cytoseal media.

### RNA sequencing

The MmuPV1 infection of BABLc *FoxN1* ^*nu/nu*^ mice was described [16]. RNA-seq analysis was performed using Illumina TrueSeq V4 chemistry on Ribo-zero total RNA isolated from uninfected- or MmuPV1-infected ear tissues using Tri-Pure Reagent (Roche). The reads trimmed by Trimmomatic (version 0.35) were mapped to mm10 (GRCm38.p3) genome by STAR aligner (version 2.4.2a) using two-pass approach. Gene-level expression quantification was performed using subread (version 1.4.6). Length and TMM normalization was performed using edgeR (version 3.12) [65] and differential expression with limma (version 3.26.3) [66]. The fold change with corresponding p-values for all genes is shown in S1 Table. The depth of coverage for selected genes was visualized using Integrated Genome Browser (https://software.broadinstitute.org/software/igv/). The original data are available at NCBI GEO repository (GSE136647).

Fresh papillomas or mock-infected ear tissues from FVB/N mice were rinsed with RNase inhibitor, snap frozen in liquid nitrogen, placed into tissueTUBE (Covaris) and pulverized by Cryoprep Pulverizer (Covaris). Total RNA was isolated by addition of 1ml or TRizol (Thermo Fisher Scientific) using RNA-binding columns (Qiagen RNA isolation kit). On column-bound RNA was treated with RQ1 RNase-free DNase (Promega) for 30 min at room temperature, washed with washing buffers, and eluted in RNase-free water. One ug of high-quality RNA (RIN>7, Agilent 2100) from each sample were used for pair-end library preparation using NEBNext Ultra Library Prep Kit for Illumina (NEB #E7770). Libraries with sizes peaked around 300bp were quantified by Qubit (Thermo Fisher) and adapter-associated fragments were quantified by NEBNext Library Quant Kit (E7630). Pooled libraries were were sequenced on Illumina HiSeq 4000. The RNA seq data were analyzed using Galaxy [67]. Low-quality ends (phred quality score threshold 20) and adapter associated reads were trimmed by Trim Galore, and reads shorter than 20 bp after trimming were discarded. Trimmed sequences were then mapped to the mouse genome (mm10) using STAR aligner. Strand (reverse) specific reads were counted by featureCounts. DESeq2 was used to analyze differentially expressed genes between groups. Gene ontology analysis was done with GeneCodis (http://genecodis.cnb.csic.es/). Gene set enrichment analysis (GSEA 3.0) was done with genes that have a human homolog (ENSEMBL). RNA-seq data has been deposited to NCBI Sequence Read Archive (SRA) (SRA accession: PRJNA560073).

### RT-qPCR

500 ng of RNA from each sample were used for cDNA synthesis using Quantitect reverse transcription kit (Qiagen). SYBR Green or TaqMan probe were then used for quantitative PCR performed on ABI 7900HT, all gene expression levels were normalized to GAPDH. The following primers were used for SYBR Green detection of mouse gene expressions: GAPDH forward 5’- CATGGCCTTCCGTGTTCCTA-3’; GAPDH reverse 5’- GCGGCACGTCAGATCCA-3’;

CXCL9 forward 5’- TCCTCTTGGGCATCATCTTCC-3’; CXCL9 reverse 5’- TTTGTAGTGGATCGTGCCTCG-3’; CXCL10 forward 5’- CCAAGTGCTGCCGTCATTTTC-3’; CXCL10 reverse 5’-GGCTCGCAGGGATGATTTCAA-3’ PD-L1 forward 5’- CCAGCCACTTCTGAGCATGA-3’; PD-L1 reverse 5’- CTTCTCTTCCCACTCACGGG-3’; IFNγ forward 5’- ACAATGAACGCTACACACTGCAT-3’; IFNγ reverse 5’- TGGCAGTAACAGCCAGAAACA-3’. The following TaqMan probes (Thermo Fisher Scientific) were used for K17 expression measurement: GAPDH (Mm99999915_g1); K17 (Mm00495207_m1).

### TCGA data

TCGA data were analyzed by http://www.cbioportal.org/. Head and neck squamous cell carcinoma (TCGA, PanCancer Atlas) dataset with 523 samples (of which 515 samples have available mRNA expression data) were selected for gene query with a Z score of 1.64. The following genes were queried for this dataset: KRT17, CXCL9, CD8A, IFNG, GZMB. Dot plots showed correlation between KRT17 and CXCL9/CD8A/IFNG for all 515 samples. Heatmap shows only samples with expression level of top 5% and bottom 5% (more than 1.64 fold of standard deviation away from the mean) in any of the queried genes.

### Statistics

All statistical analyses were done with Graphpad Prism. Two-way ANOVA was used for statistical comparison when two variables (infection and genotype) were involved. For single variable experiments, t test or one-way ANOVA was used for statistical comparison as indicated.

## Supporting information

S1 Table Differential expression of mouse genes induced in ear MmuPV-1 papillomas (versus the uninfected normal ear tissues). The original data are available at GEO repository (Accession number GSE136647). Related to Fig 1A.
(XLSX)

S2 Table Differential expression of mouse genes in MmuPV-1 papillomas arising in K17KO mice versus WT mice. Related to Fig 3B.
(XLSX)

S3 Table Gene ontology analysis for differentially expressed genes in K17KO papilloma versus WT papilloma. Related to Table 1 and Fig 3C.
(XLSX)

S4 Table Gene set list of GSEA analysis for genes homologous to human genes. Related to Fig 3D.
(XLSX)

S5 Table Gene list of top two enriched gene sets by GSEA. Related to Fig 3D.
(XLSX)

S1 Fig Upregulation of stress keratin 16, 6b and 6a in MmuPV1 papillomas. Related to Fig 1A. Papillomas-bearing and mock-infected ears harvested at 6 months post infection from BABLc *FoxN1* nude mice were analyzed by RNA-seq. RNA-seq reads mapped to corresponding Krt16, 6b or 6a genes in each sample were visualized by IGV with indicated scale.
(PDF)

S2 Fig T cells were depleted in circulating blood as well as in ear papilloma following scheduled depletion antibody injection. Related to Fig 2. A) Flow cytometry analysis of CD4 and CD8 staining of blood collected by submandibular bleeding 5 weeks post infection, from CD4+CD8 depleted K17KO mice (top) or isotype control injected K17KO mice (bottom). Cells shown were pre-gated on single live CD45+ cells. Three representative mice are shown for each group; B) Flow cytometry analysis of CD4 and CD8 staining of ear papilloma from CD4+CD8 depleted K17KO mice (6-week papilloma) and untreated K17KO mice (6-week papilloma). Three mice are shown for each group. For T cell depletion, 100 ug of anti-CD4 (BioXCell, clone GK1.5) and 100 ug of anti-CD8 antibody (BioXCell, clone 2.43) or 100 ug of isotype control (BioXCell, Rat IgG2b, κ) was delivered by intraperitoneal injection twice weekly, starting 4 days before MmuPV1 infection throughout the study. For detection of CD4 and CD8 depletion, CD8a FITC (Tonbo ebioscience, clone 53–6.7), CD4 PE (Tonbo ebioscience, clone RM4-5) were used for flow cutometry.
(PDF)

S3 Fig A variety of immune cell populations were found in MmuPV1 infection-induced ear papilloma. Related to Fig 4A. A) Gating example for flow cytometry analysis on MmuPV1-induced papilloma sample. Only single live cells were included in quantification analysis. B) Percentage of live cells for each immune cell population based on flow cytometry analysis from MmuPV1-infected lesions at 7 weeks post-infection in FVB/N mice (1x10^9^ MmuPV1 VGE infected per site). All groups were compared to normal ear by one-way ANOVA Dunnett's multiple comparisons test. *p<0.05; **p<0.01; ***p<0.005; ns = not significant. C) Immunofluorescent staining for CD8 (green) and CD4 (green), keratin 14 (K14, red) and DAPI (blue) in MmuPV1-induced papillomas (top) and adjacent normal epithelial tissue (bottom). Scale bar in top row also applies to bottom row.
(PDF)

S4 Fig No significant difference in papilloma infiltrating CD11b^+^Gr1^+^ or F4/80^+^ cells or in Th1 or Th2 population frequency within papilloma-draining lymph nodes between K17KO and WT FVB/N mice. Related to Fig 4A. A) Papillomas harvested at 4 weeks post infection were analyzed for F4/80, CD11b and Gr1 staining. B) Papilloma draining LN (PD-LN) were harvested at 4 weeks post infection and cultured with PMA/Ion and Golgi stop for 16 hours. Intracellular IFNγ, IL4 and IL17 were measured by flow cytometry.
(PDF)

S5 Fig CXCR3+ T cells were undetectable in circulating blood following anti-CXCR3 i.p. injection. Related to Fig 5. Flow cytometry analysis of circulating blood showed undetectable level of CXCR3 using the same clone of anti-CXCR3 antibody. CXCR3, CD45, and CD8 staining of blood collected by submandibular bleeding 6 weeks post infection, from anti-CXCR3 treated K17KO mice (top) or isotype control injected K17KO mice (bottom). Three representative animals are shown. For CXCR3 blocking, 400ug of anti-CXCR3 (BioXCell, clone CXCR3-173) or isotype control antibody (BioXCell, Armenian Hamster IgG) was delivered i.p. three times a week, starting 4 days before MmuPV1 infection, throughout the study. This anti-CXCR3 clone is a well-established blocking antibody for CXCR3 in mouse studies [38, 62]. For detection of CXCR3 blocking in mice, the same clone was used (Biolegend, clone CXCR3-173).
(PDF)

S6 Fig Increased mRNA level of *KRT17* was correlated with decreased level of *CXCL9* and *CD8A* in patient head and neck cancer samples. A) Dot plot of N = 515 patient RNA-seq data. There is a weak correlation between K17 expression and *CXCL9*, *CD8A* and *IFNG* expression. B) Heatmap of *KRT17*, *CXCL9*, *CD8A*, *IFNG* and *GZMB* of patient samples with a Z-score equal to or above 1.64 in any of the queried genes.
(PDF)

S7 Fig Increased circulating CD11b^+^Gr1 high cells in blood of papilloma-bearing K17KO mice 3 months and 6 months post-infection comparing to K17KO that completely cleared papilloma. Mouse ears were infected with 2x10^8^ VGE MmuPV1. Blood were collected by submandibular bleeding at 3 months post infection from papilloma-persistent K17KO mice and papilloma-free mice and prepared to CD11b and Gr1 staining.
(PDF)

S8 Fig *In vitro* infectivity quantification of two preparations of MmuPV1 used. JB6 clone41-5a cells were at 10^5 cells per well in 6-well plate 24 hours before infection with MmuPV1 Prep#1 and Prep#2 with indicated VGE. Fourty-eight hours after infection, RNA were harvested from each well and E1^E4 transcript and GAPDH transcript levels were detected by qRT-PCR. Log (agonist) vs. response (three parameters) was used to best fit both curves.
(PDF)
