# Supplementary figures and images for: Stress keratin 17 enhances papillomavirus infection-induced disease by downregulating T cell recruitment
Source: PLoS Pathog. 2020 Jan 22;16(1):e1008206. doi: 10.1371/journal.ppat.1008206 (PMC6975545; doi:10.1371/journal.ppat.1008206)

### Krt16

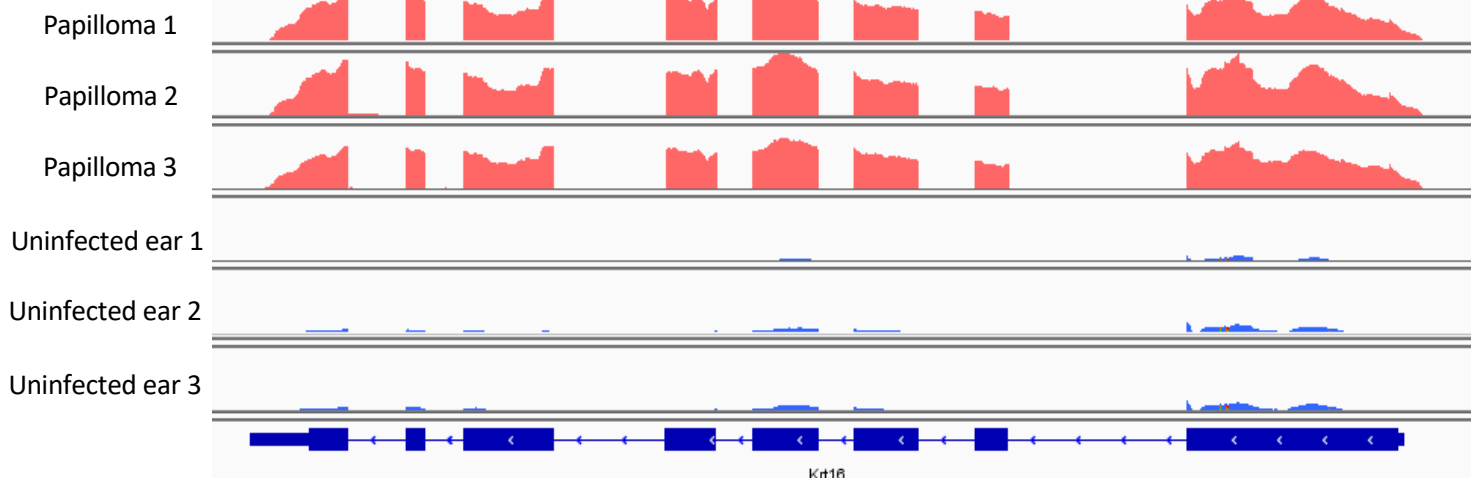

### Krt6b

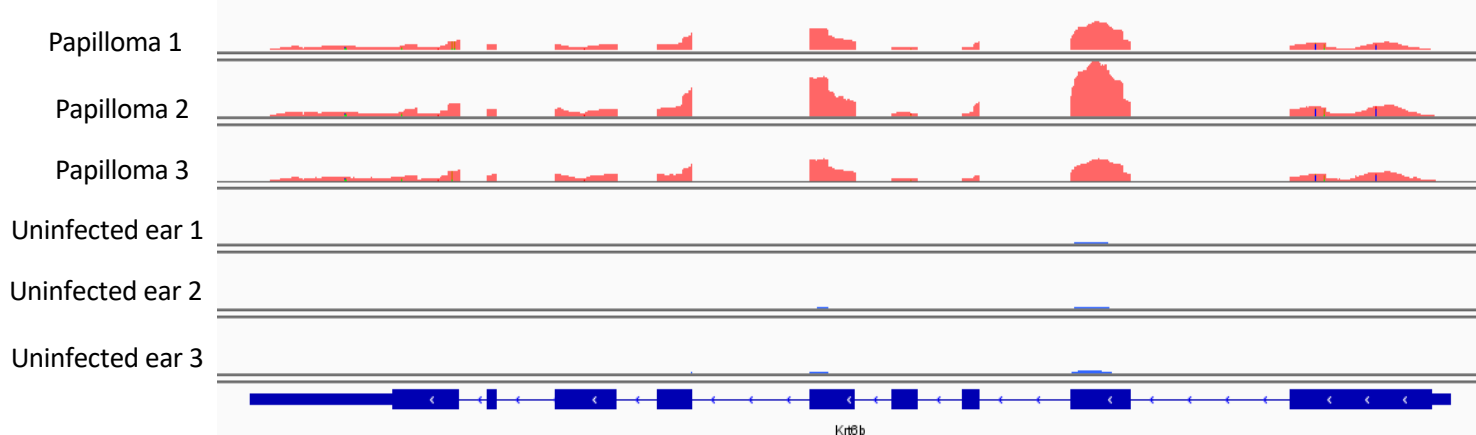

### Krt6a

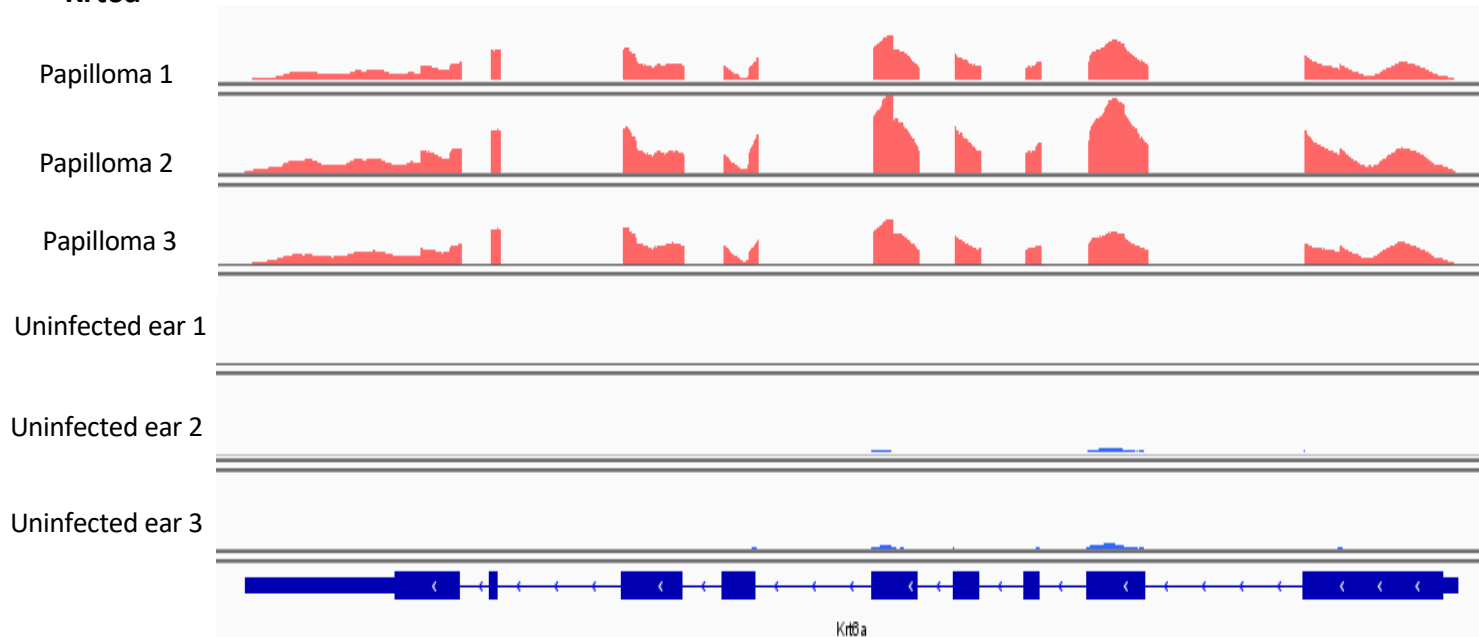

Supplement: S1 Fig — Related to Fig 1A. Papillomas-bearing and mock-infected ears harvested at 6 months post infection from BABLc FoxN1 nude mice were analyzed by RNA-seq. RNA-seq reads mapped to corresponding Krt16, 6b or 6a genes in each sample were visualized by IGV with indicated scale. (PDF) [file ppat.1008206.s006.pdf]

**A**

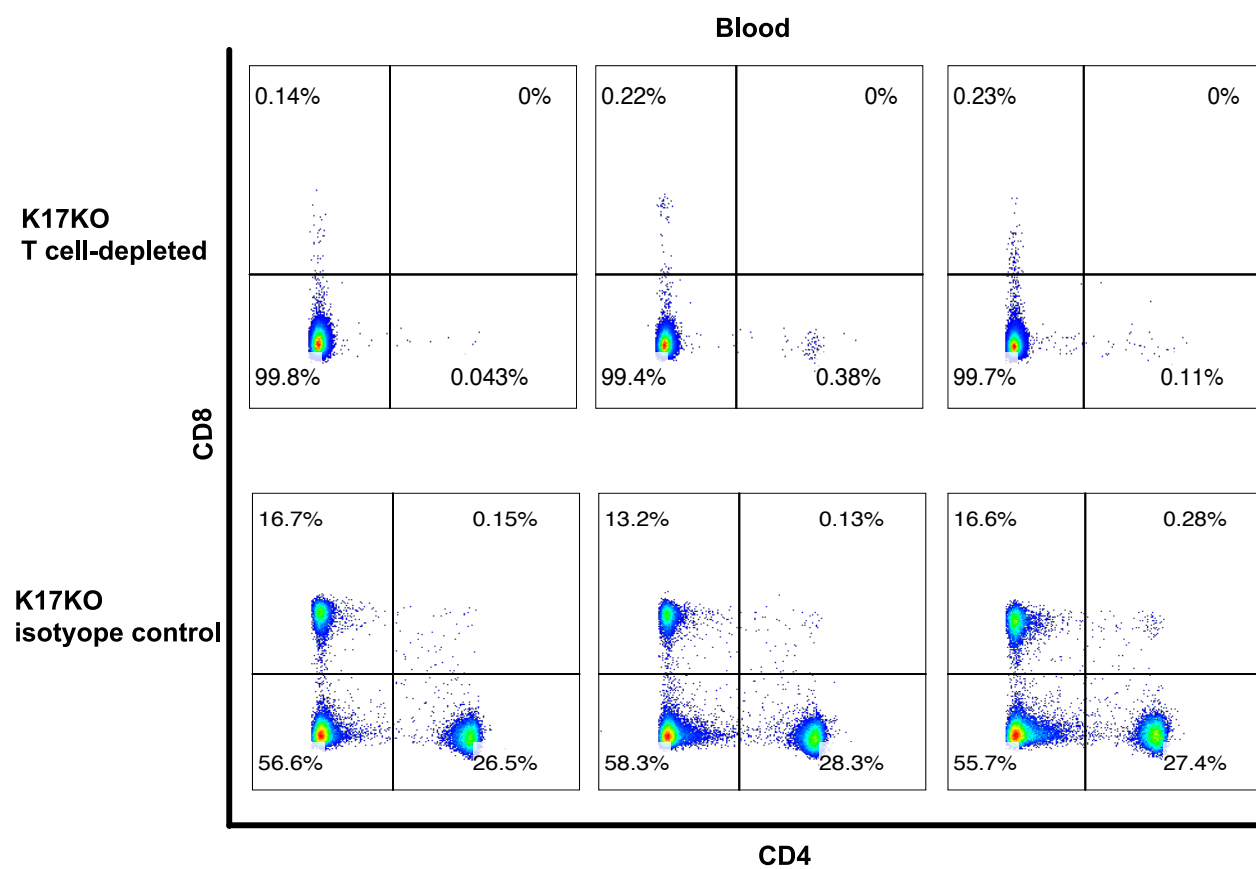

**B**

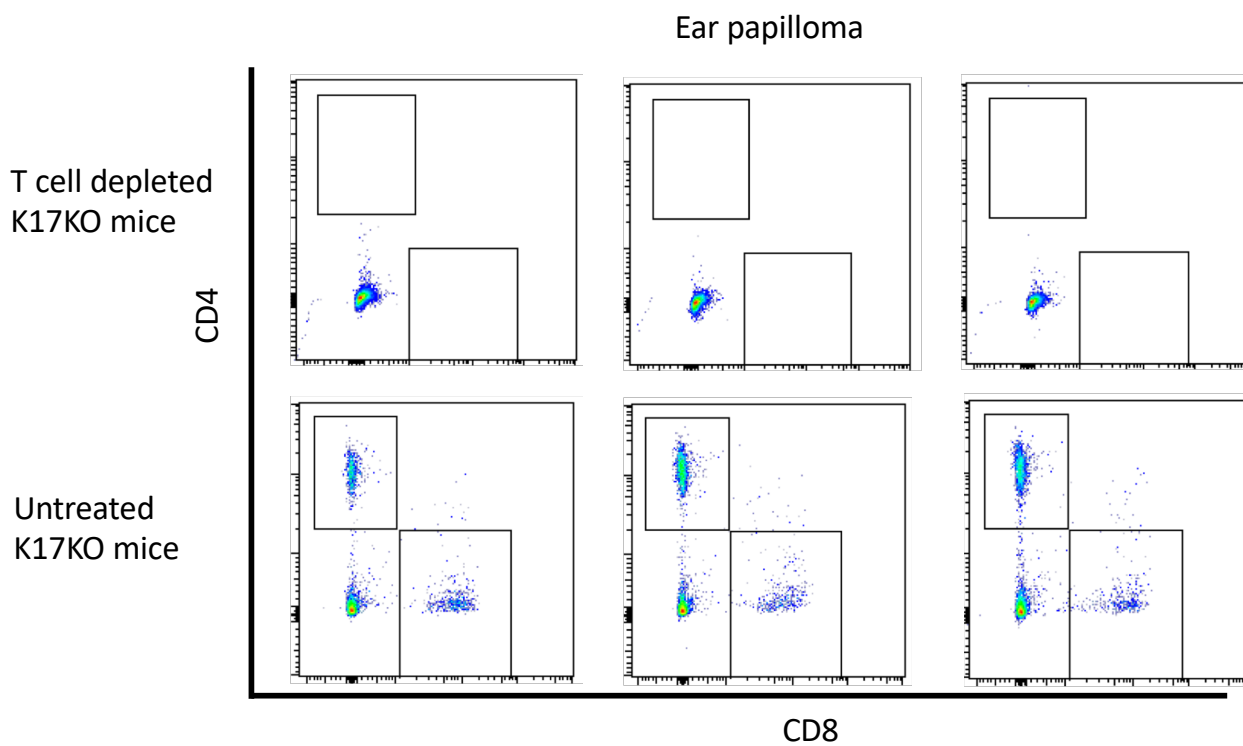

Supplement: S2 Fig — Related to Fig 2. A) Flow cytometry analysis of CD4 and CD8 staining of blood collected by submandibular bleeding 5 weeks post infection, from CD4+CD8 depleted K17KO mice (top) or isotype control injected K17KO mice (bottom). Cells shown were pre-gated on single live CD45+ cells. Three representative mice are shown for each group; B) Flow cytometry analysis of CD4 and CD8 staining of ear papilloma from CD4+CD8 depleted K17KO mice (6-week papilloma) and untreated K17KO mice (6-week papilloma). Three mice are shown for each group. For T cell depletion, 100 ug of anti-CD4 (BioXCell, clone GK1.5) and 100 ug of anti-CD8 antibody (BioXCell, clone 2.43) or 100 ug of isotype control (BioXCell, Rat IgG2b, κ) was delivered by intraperitoneal injection twice weekly, starting 4 days before MmuPV1 infection throughout the study. For detection of CD4 and CD8 depletion, CD8a FITC (Tonbo ebioscience, clone 53–6.7), CD4 PE (Tonbo ebioscience, clone RM4-5) were used for flow cutometry. (PDF) [file ppat.1008206.s007.pdf]

A.

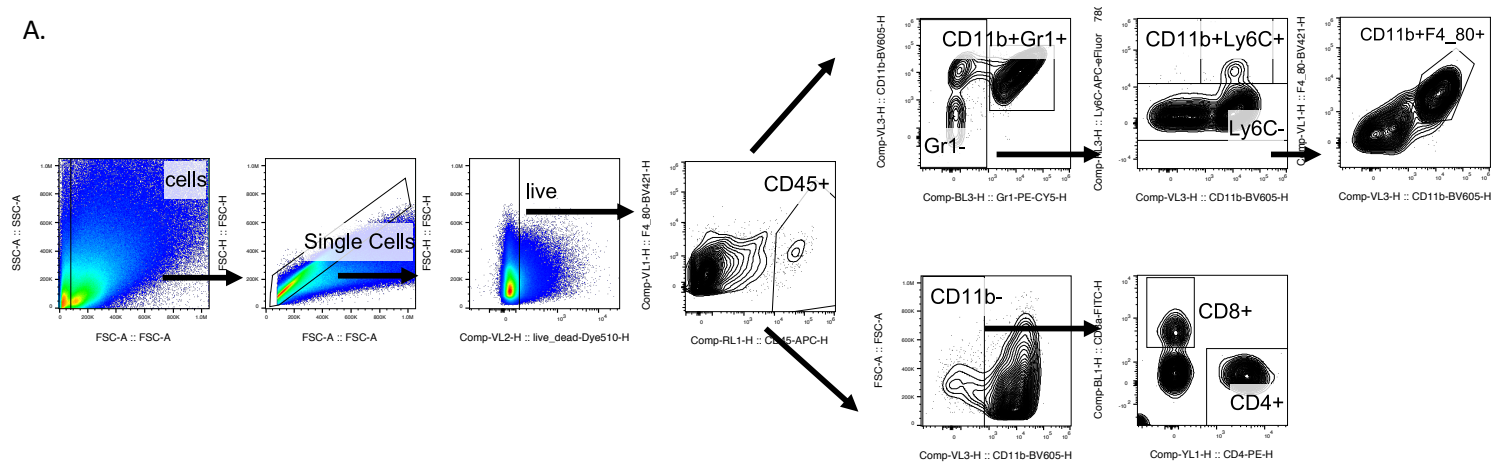

B.

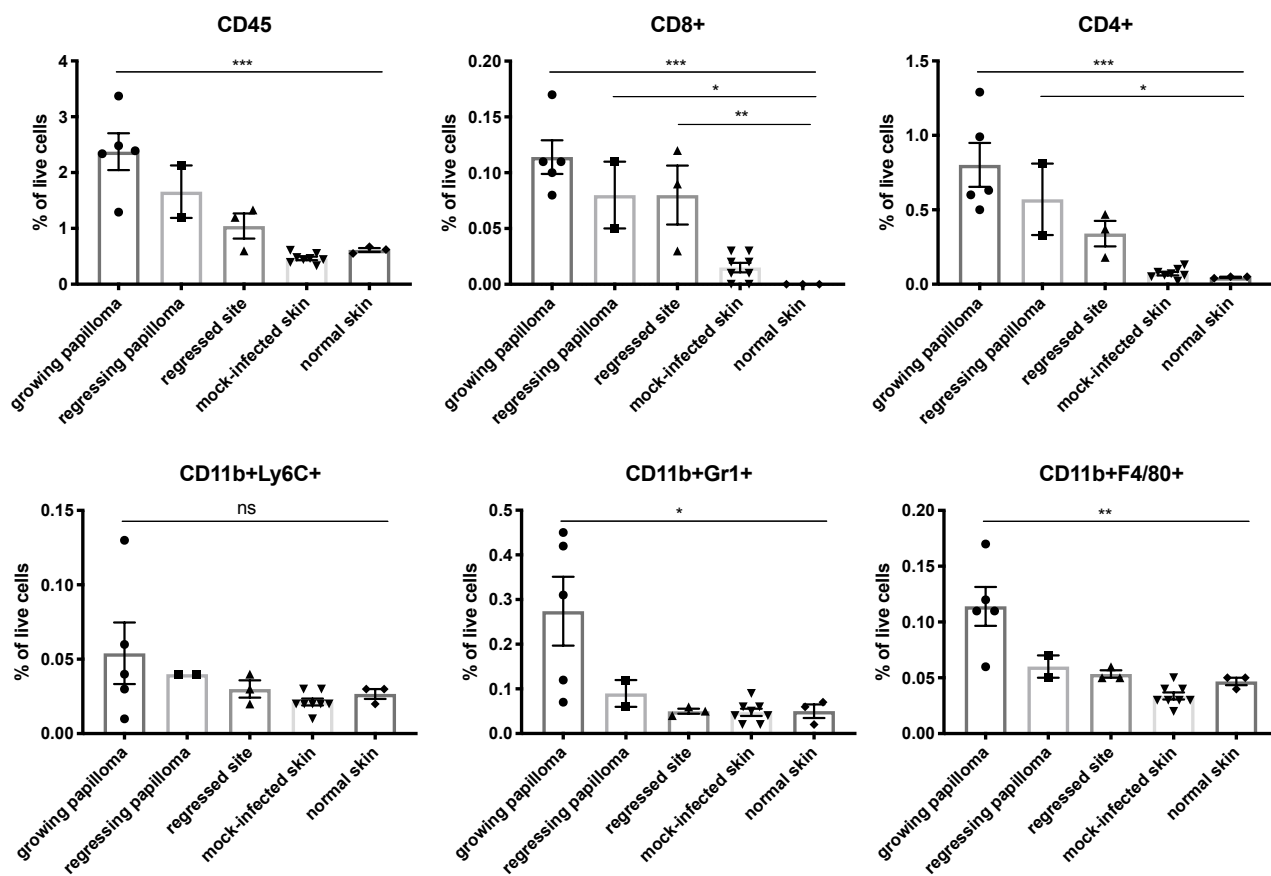

C.

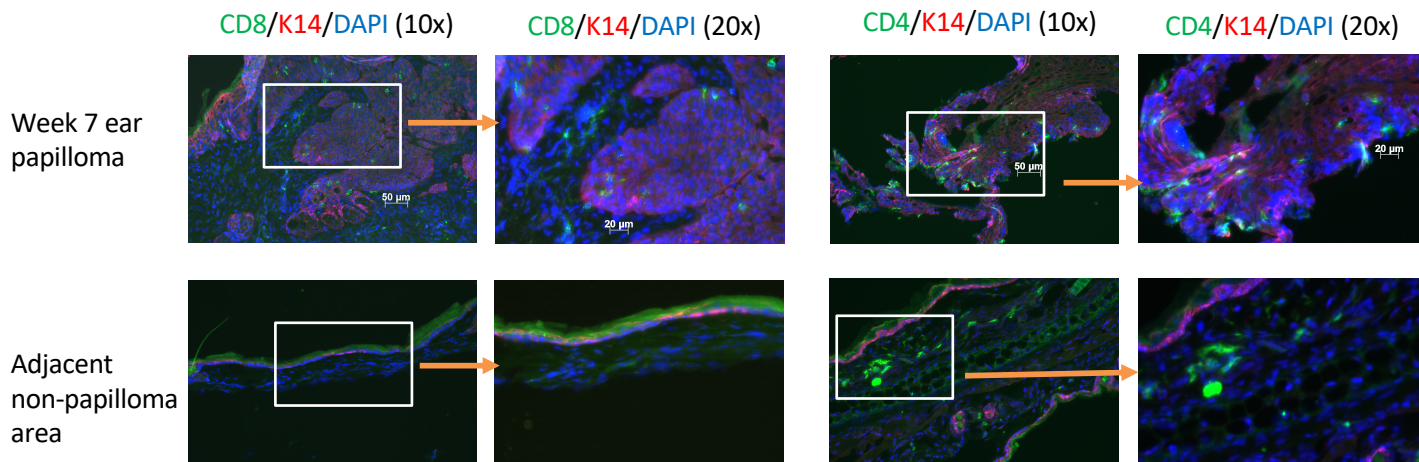

Supplement: S3 Fig — Related to Fig 4A. A) Gating example for flow cytometry analysis on MmuPV1-induced papilloma sample. Only single live cells were included in quantification analysis. B) Percentage of live cells for each immune cell population based on flow cytometry analysis from MmuPV1-infected lesions at 7 weeks post-infection in FVB/N mice (1x10^9^ MmuPV1 VGE infected per site). All groups were compared to normal ear by one-way ANOVA Dunnett's multiple comparisons test. *p<0.05; **p<0.01; ***p<0.005; ns = not significant. C) Immunofluorescent staining for CD8 (green) and CD4 (green), keratin 14 (K14, red) and DAPI (blue) in MmuPV1-induced papillomas (top) and adjacent normal epithelial tissue (bottom). Scale bar in top row also applies to bottom row. (PDF) [file ppat.1008206.s008.pdf]

A.

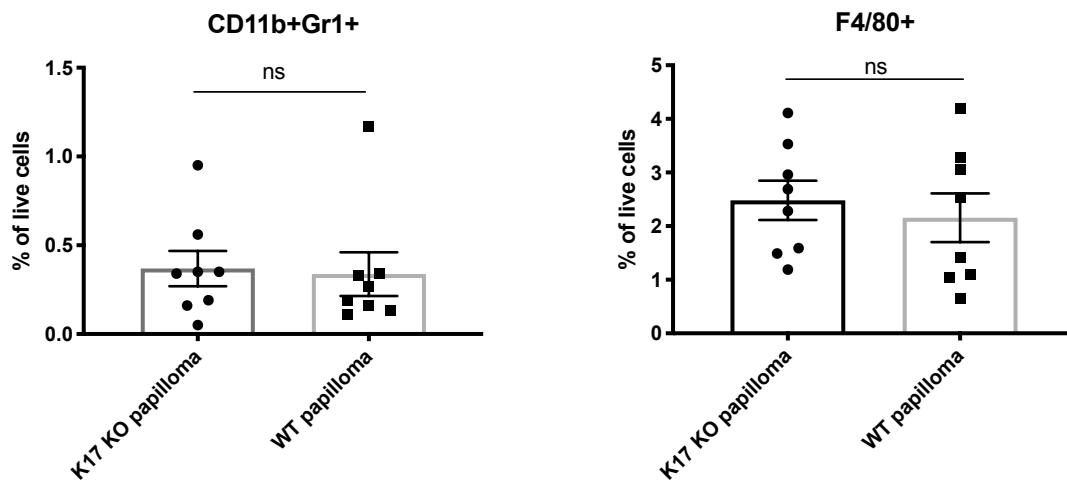

B.

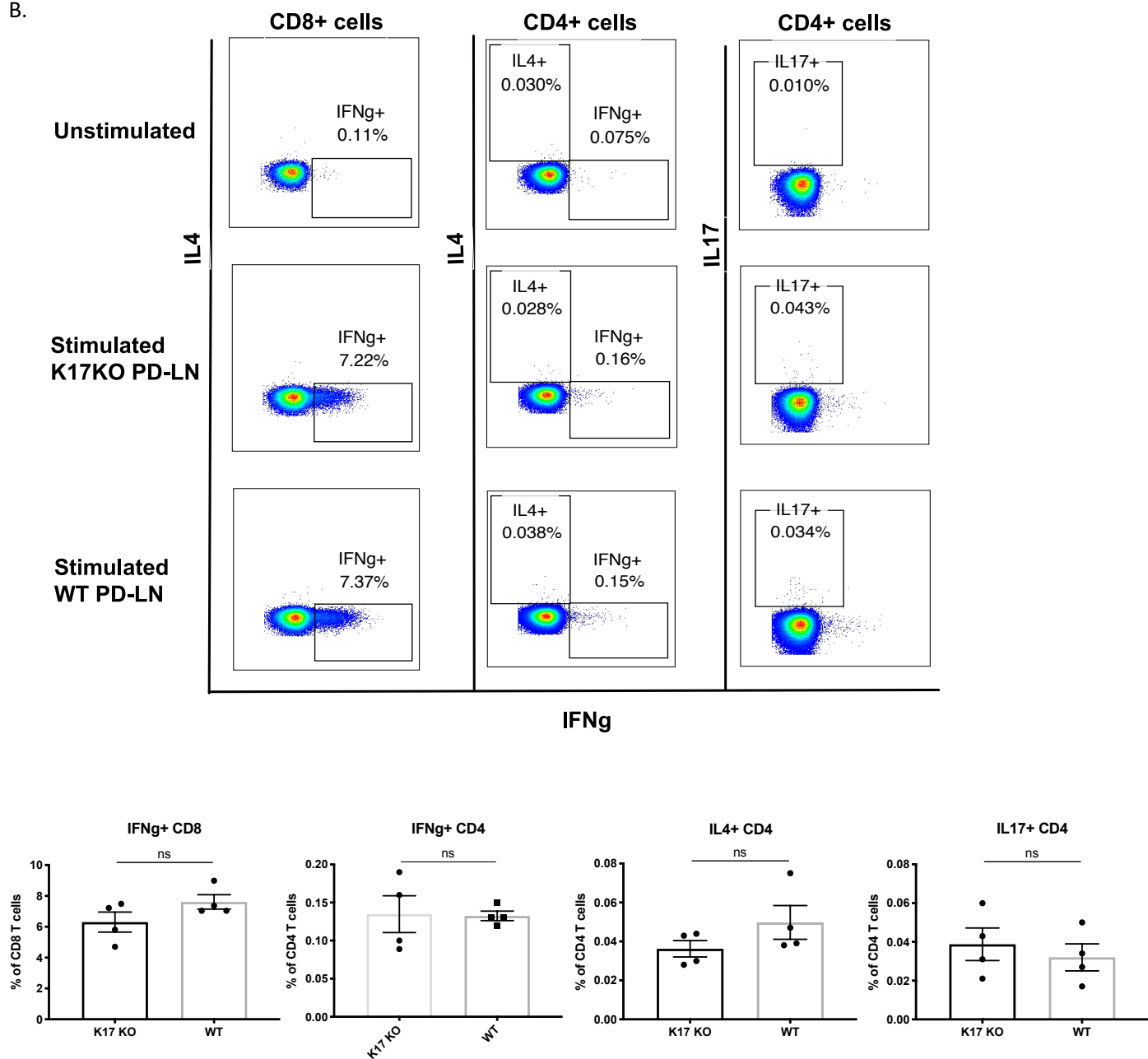

Supplement: S4 Fig — Related to Fig 4A. A) Papillomas harvested at 4 weeks post infection were analyzed for F4/80, CD11b and Gr1 staining. B) Papilloma draining LN (PD-LN) were harvested at 4 weeks post infection and cultured with PMA/Ion and Golgi stop for 16 hours. Intracellular IFNγ, IL4 and IL17 were measured by flow cytometry. (PDF) [file ppat.1008206.s009.pdf]

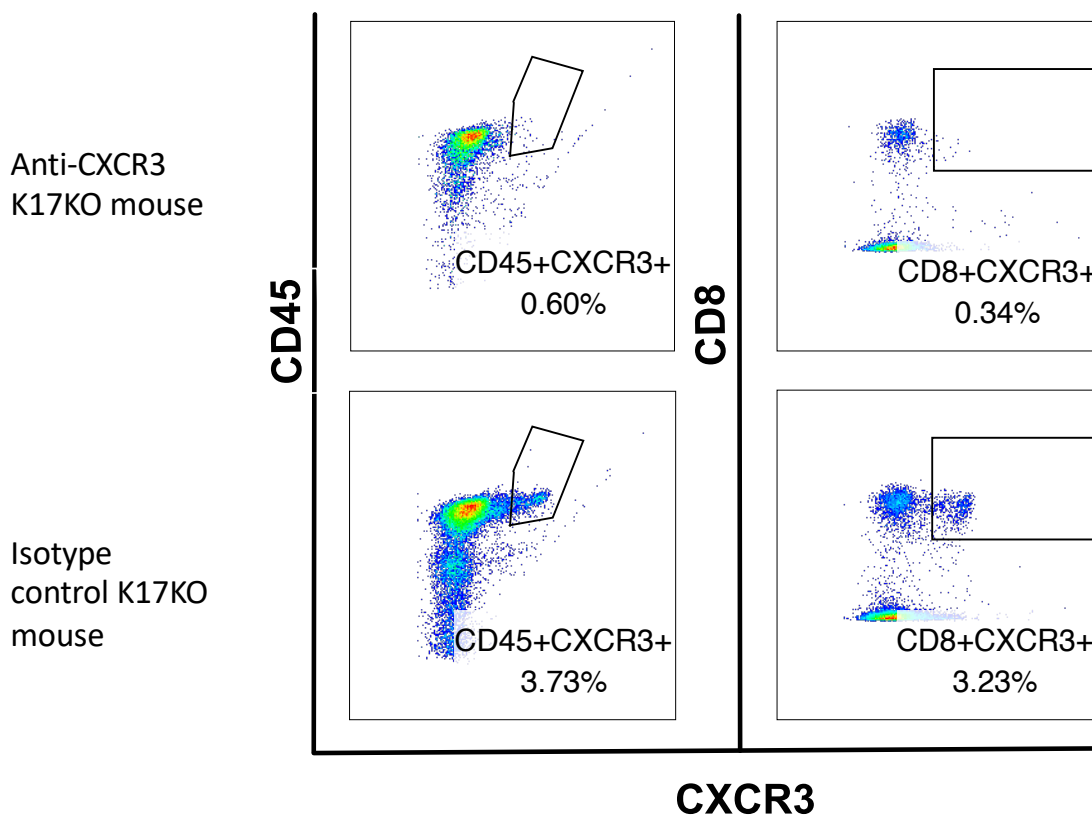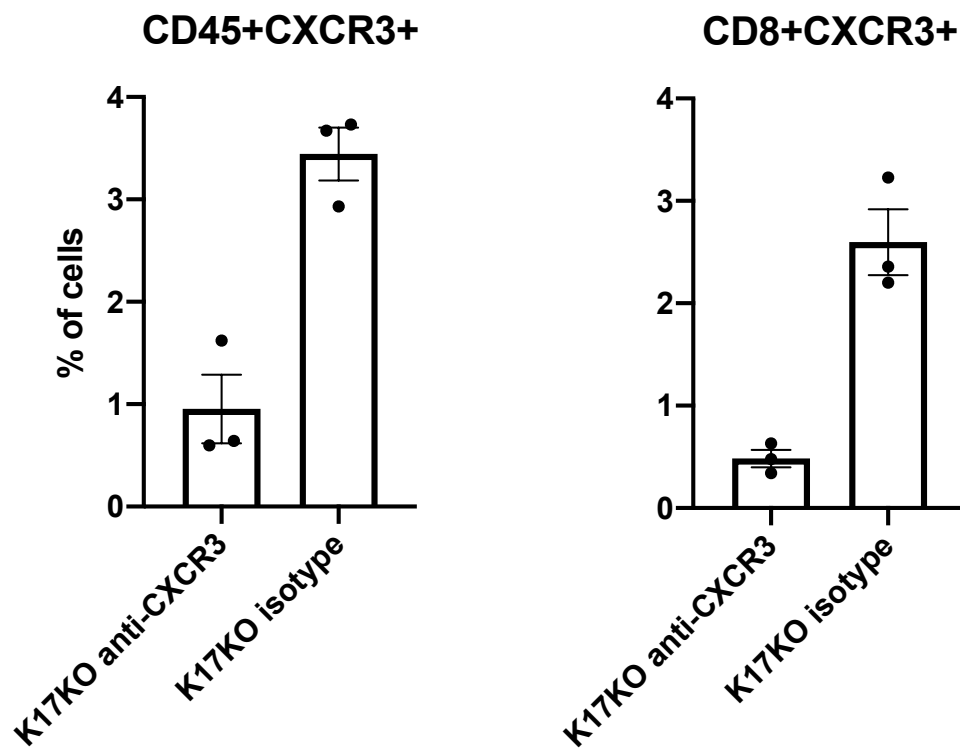

Supplement: S5 Fig — Related to Fig 5. Flow cytometry analysis of circulating blood showed undetectable level of CXCR3 using the same clone of anti-CXCR3 antibody. CXCR3, CD45, and CD8 staining of blood collected by submandibular bleeding 6 weeks post infection, from anti-CXCR3 treated K17KO mice (top) or isotype control injected K17KO mice (bottom). Three representative animals are shown. For CXCR3 blocking, 400ug of anti-CXCR3 (BioXCell, clone CXCR3-173) or isotype control antibody (BioXCell, Armenian Hamster IgG) was delivered i.p. three times a week, starting 4 days before MmuPV1 infection, throughout the study. This anti-CXCR3 clone is a well-established blocking antibody for CXCR3 in mouse studies [38, 62]. For detection of CXCR3 blocking in mice, the same clone was used (Biolegend, clone CXCR3-173). (PDF) [file ppat.1008206.s010.pdf]

A.

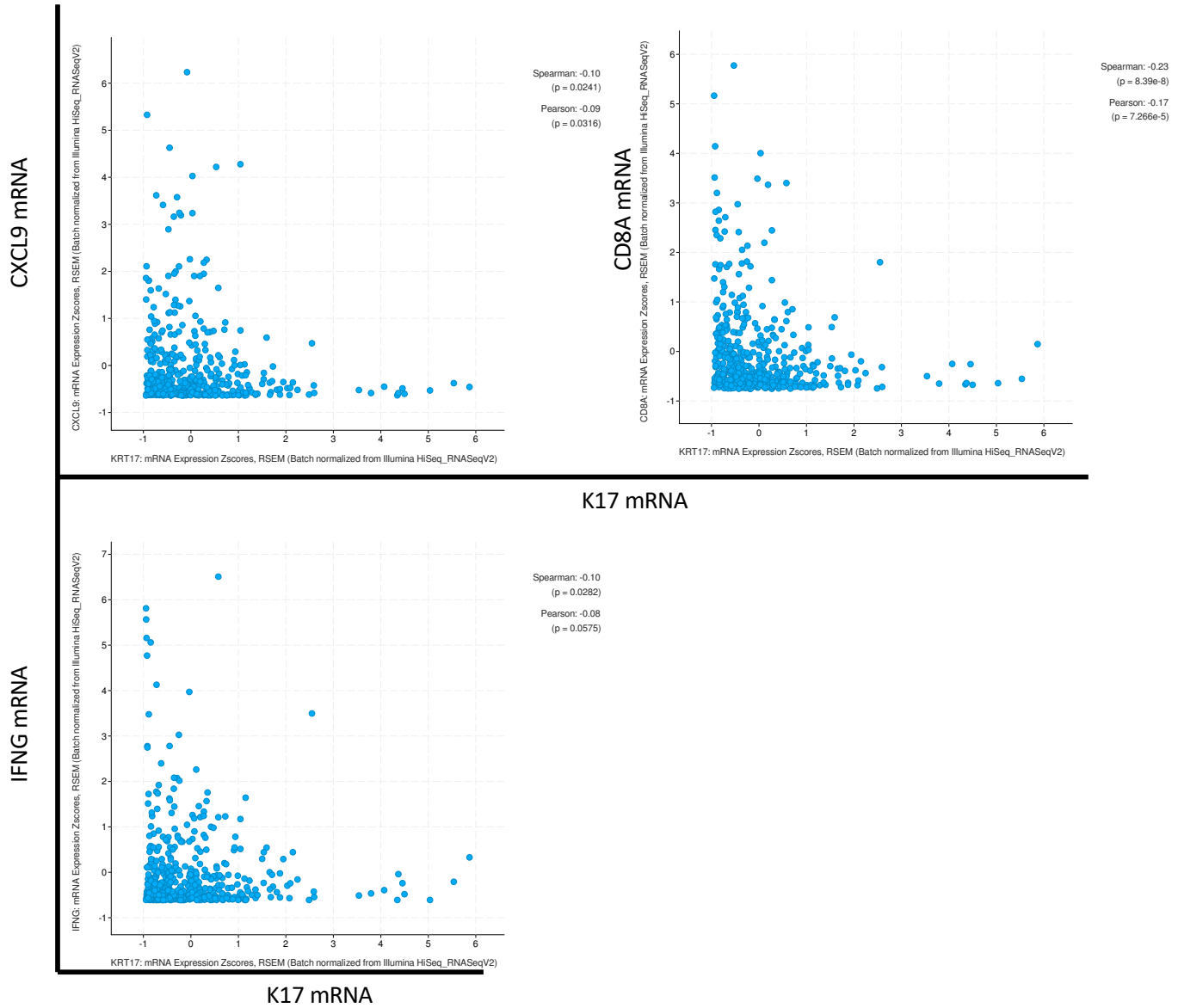

B.

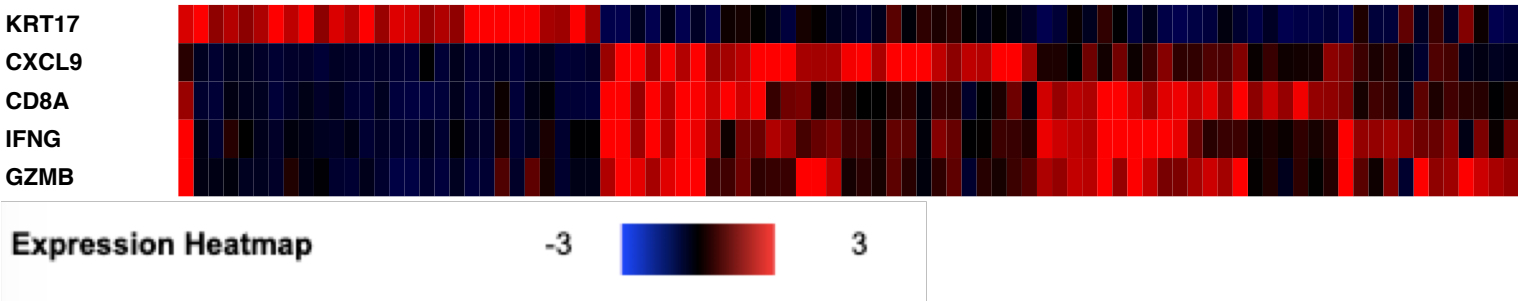

Supplement: S6 Fig — A) Dot plot of N = 515 patient RNA-seq data. There is a weak correlation between K17 expression and CXCL9, CD8A and IFNG expression. B) Heatmap of KRT17, CXCL9, CD8A, IFNG and GZMB of patient samples with a Z-score equal to or above 1.64 in any of the queried genes. (PDF) [file ppat.1008206.s011.pdf]

Blood 3 months post infection

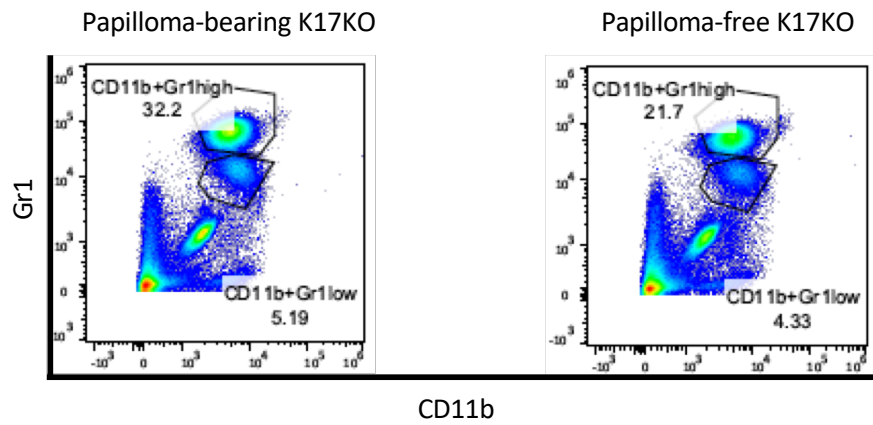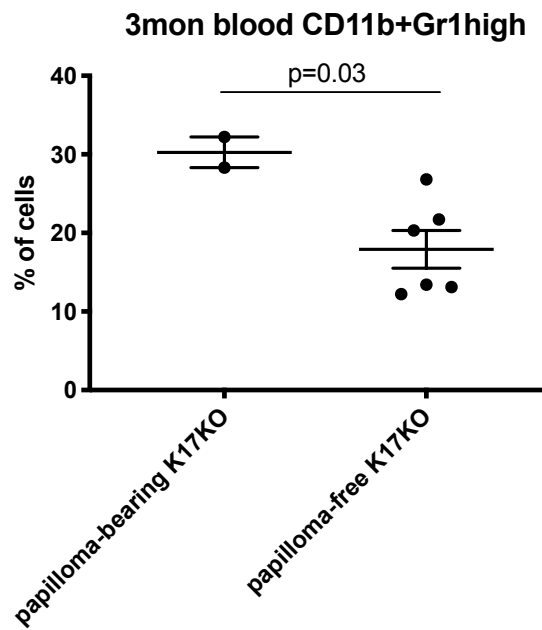

Supplement: S7 Fig — Mouse ears were infected with 2x10^8^ VGE MmuPV1. Blood were collected by submandibular bleeding at 3 months post infection from papilloma-persistent K17KO mice and papilloma-free mice and prepared to CD11b and Gr1 staining. (PDF) [file ppat.1008206.s012.pdf]

*In vitro* infectivity

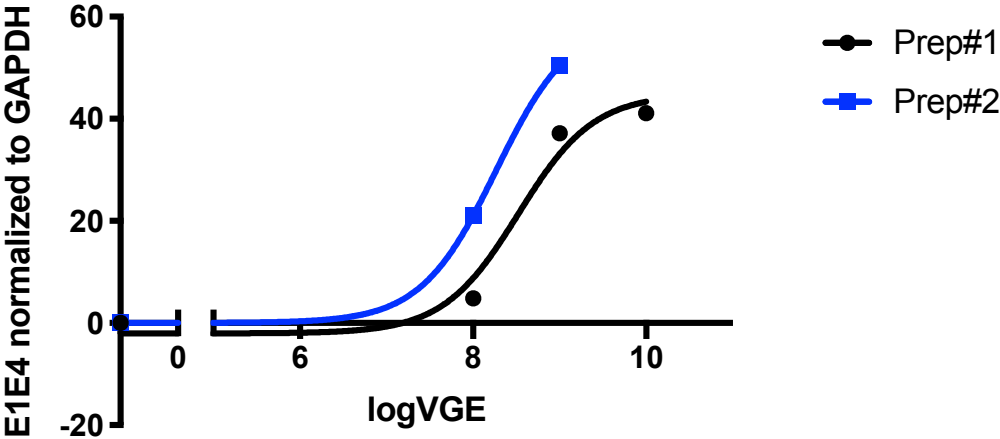

Supplement: S8 Fig — JB6 clone41-5a cells were at 10^5 cells per well in 6-well plate 24 hours before infection with MmuPV1 Prep#1 and Prep#2 with indicated VGE. Fourty-eight hours after infection, RNA were harvested from each well and E1^E4 transcript and GAPDH transcript levels were detected by qRT-PCR. Log (agonist) vs. response (three parameters) was used to best fit both curves. (PDF) [file ppat.1008206.s013.pdf]
